# Revision of the Hawaiian psyllid genus *Swezeyana*, with descriptions of seven new species (Hemiptera, Psylloidea, Triozidae)

**DOI:** 10.3897/zookeys.758.23019

**Published:** 2018-05-15

**Authors:** Diana M. Percy

**Affiliations:** 1 Department of Life Sciences, Natural History Museum, Cromwell Road, London, UK; 2 and Department of Botany, University of British Columbia, University Boulevard, Vancouver, BC, Canada

**Keywords:** jumping plant lice, mitochondrial DNA barcode, *Planchonella*, Sapotaceae, taxonomy

## Abstract

The endemic Hawaiian genus *Swezeyana* Caldwell, 1940 is highly distinctive due to the extremely long genal processes. In addition, some of the immatures are ornamented with extraordinary tubercles and tentacles. Two *Swezeyana* species are redescribed, and seven new species are described, bringing the total number of species in the genus to nine. All species are hosted by a single, endemic host plant, *Planchonella sandwicensis* (Sapotaceae), which is distributed across all major islands in the archipelago. The majority of *Swezeyana* species are single island endemics. A sister taxon pair is found sympatrically on the same individual plants on Kauai, and putative sister or at least closely related species are also found sympatrically on Oahu and Hawaii, suggesting these taxa may have diversified in sympatry. However, there is no observed ecological niche partitioning, despite some striking morphological diversity, as all *Swezeyana* species have free-living immatures that are found on the leaf surface, and therefore no apparent biological shifts are coincident with occupying the same host plant. Two species groups are represented by strikingly different female terminalia structure and endoskeletal development, although ovipositor structure is very similar between the two groups. Mitochondrial DNA barcodes (COI and cytB) are provided for eight of the nine species. A phylogenetic analysis of the mitochondrial barcode regions indicates species relationships within *Swezeyana* and provides a comparison of genetic divergence with other Hawaiian endemic genera.

## Introduction

The Hawaiian Islands are one of the most isolated terrestrial landscapes on earth with high levels of endemism reflecting both limited immigration and in situ diversification (Gillespie et al. 2012), Because these islands are relatively young geologically, they provide a snap shot of evolutionary processes occurring early in lineage diversification that are often obscured over time in older landscapes. We can witness processes such as early burst adaptive radiation for which there is rarely evidence in continental landscapes (Losos and Ricklefs 2009). One can also see these patterns replicated across different islands in an archipelago adding support to interpretations of early speciation processes (Gillespie 2004).

The Hawaiian psyllid fauna is relatively well known compared to other tropical faunas (Zimmerman 1948). The native species and most of the described genera are endemic with a number of exemplary cases of adaptive radiations on endemic host plants (e.g., *Pariaconus* Enderlein, 1926 on *Metrosideros* (Myrtaceae), *Hevaheva* Kirkaldy, 1902 on *Melicope* (Rutaceae), and *Megatrioza* Crawford, 1915 on *Pritchardia* (Arecaceae)) (Zimmerman 1948, Uchida and Beardsley 1988, Percy 2017a). Increasingly, molecular data on these species radiations is contributing additional information required to interpret evolutionary processes, but there remains considerably more work to do to reveal whether common dynamics are driving evolution across these different psyllid groups, as well as to determine the origins of the closest relatives outside the Hawaiian Islands (Percy 2017a). In addition, there are pressing conservation issues related to documenting psyllid diversity and species that are host specific on native plants in a rapidly eroding and threatened native flora (Percy 2017b).

*Swezeyana* Caldwell, 1940 is an endemic Hawaiian genus with two previously described species, *Swezeyana elongagena* Caldwell, 1940 and *Swezeyana reticulata* Caldwell, 1940, which are here redescribed and seven new species are added. All *Swezeyana* species occur on a single, endemic host plant, *Planchonella sandwicensis* (Sapotaceae). *Planchonella sandwicensis* is scattered in abundance, and only occasionally locally common in some areas of the archipelago. However, *Swezeyana* species are only rarely encountered, and where found, abundances are usually low with only a few individuals collected; although in a short note on *Swezeyana*, Tuthill (1966) remarked that immatures of two species were observed to be abundant on Maui in 1965, but he also remarked that this represented the first collection of the genus since the type material was collected in the 1920s and 1930s (Caldwell 1940). Immatures and adults are found on the leaf surface, and all species for which the biology is known have free-living immatures. There is relatively little morphotypic variation in the host plant across the different islands, compared, for instance, to the morphotypic variation found in the host plant of *Pariaconus*, *Metrosideros polymorpha* (Percy et al. 2008, Percy 2017a). The absence of observed differences in immature biologies and habitats, and the lack of host variation make it therefore difficult to interpret the drivers of the often striking morphological diversity, particularly in the immatures (Tuthill 1966).

The adult morphology is most obviously characterized by the extremely long genal processes and some unusual structural features of the fore wing. The fore wing has a more or less extended “pseudopterostigma” which appears as a thickened anterior fore margin extending from a position parallel to the trifurcation of R, M and Cu_1_ to approximately 1/3 to 1/2 the length of vein Rs. In reference to this feature, Zimmerman (1948) referred to the radius (R) becoming “obsolete” beyond the origin of vein Rs. In addition, often present are few to many partial or fully developed cross veins in cell r_1_ traversing between vein Rs and the anterior wing margin. Further adult variation is provided by the presence or absence of wing patterns and the striking differences in the structure of the female terminalia.

The objective of this study is to detail the diversification and distribution of this unusual and uncommon Hawaiian endemic genus, which provides yet another example of in situ psyllid diversification within the Hawaiian Islands. Understanding parallel processes of potential sympatric diversification in *Swezeyana* will contribute to our knowledge of speciation processes in Psylloidea more broadly.

## Materials and methods

Field collections were made in May–July 2002, August 2003, February 2011, May–July 2014. Adults were preserved in 95% ethanol. For morphological examination, ethanol-preserved material was macerated and cleared in 10% potassium hydroxide followed by clove oil, and slide mounted in Canada balsam as described in Hodkinson and White (1979). Morphological terminology follows Hodkinson and White (1979), Hollis (1984), and Percy (2003a, 2017a). The DNA barcodes provided here were sequenced from two mitochondrial gene regions, cytochrome oxidase subunit 1 (COI), and cytochrome B (cytB). DNA was obtained from material preserved in ethanol, and protocols for DNA extraction, polymerase chain reaction and sequencing follow those described in Percy (2003b); polymerase chain reaction primers for COI and cytB, respectively, are given in Simon et al. (1994) and Timmermans et al. (2010), see also Percy et al. (2018). Genetic distances reported here and the phylogenetic analysis with bootstrap support (1000 replicates) were obtained using neighbour-joining (NJ) analyses with uncorrected (p) distances in PAUP* (Swofford 2003). For comparison of topology and node support the following analyses were run on the CIPRES Science Gateway (Stamatakis 2014, Miller et al. 2010): a Maximum likelihood (ML) analysis using RAxML (v. 8.2.4) with GTRCAT, 1000 rapid bootstraps, and Gamma optimization of tree space; and a Bayesian analysis using MrBayes (v. 3.2.6) with two independent runs with four coupled MCMC chains run for 20 million generations, sampling every 1000^th^ generation, and visualized using a 50% majority-rule consensus tree with 25% of topologies discarded as burn-in (Ronquist and Huelsenbeck 2003).The molecular analysis includes eight of the nine *Swezeyana* species and 16 other taxa from Triozidae, mostly representing other Hawaiian genera for comparative divergence analysis; and one species from Carsidaridae as an outgroup (*Mesohomotoma hibisci* (Froggatt, 1901)) (Table 1). The DNA sequences are deposited in GenBank. Type material is deposited in the Natural History Museum, London, UK (BMNH).

**Table 1.** Additional taxa sampled for the mitochondrial DNA analysis, with GenBank accession numbers.

Species	Locality	GenBank COI/cytB (publication)
**Family: Carsidaridae**		
*Mesohomotoma hibisci* (Froggatt, 1901)	Society Islands (Moorea)	KY294174/KY294658 (Percy 2017a)
	Society Islands (Raiatea)	KY294171/KY294655 (Percy 2017a)
	New Caledonia	KY294170/KY294654 (Percy 2017a)
	Singapore	KY294176/KY294660 (Percy 2017a)
**Family: Triozidae**		
*Anomocephala unica* Tuthill, 1942	Austral Islands (Rapa)	KY293698/KY294177 (Percy 2017a)
*Bactericera cockerelli* (Šulc, 1909)	California	KY011201/KY011296 (Percy 2017a)
*Hemischizocranium aloha* (Caldwell, 1940)	Hawaiian Islands (Kauai)	MG988755/MG989062 (this study)
*Hemischizocranium bessi* Tuthill, 1956	Hawaiian Islands (Hawaii)	MG988756/MG989063 (this study)
*Hevaheva maculata* Caldwell, 1940	Hawaiian Islands (Kauai)	KY293702/KY294181 (Percy 2017a)
*Hevaheva minuta* Crawford, 1925	Hawaiian Islands (Kauai)	KY293703/KY294182 (Percy 2017a)
*Hevaheva perkinsi* Kirkaldy, 1902	Hawaiian Islands (Oahu)	KY293704/KY294183 (Percy 2017a)
*Hevaheva silvestris* Kirkaldy, 1908	Hawaiian Islands (Oahu)	KY293705/KY294184 (Percy 2017a)
*Pariaconus iolani* (Kirkaldy, 1902)	Hawaiian Islands (Kauai)	KY293820/KY294297 (Percy 2017a)
*Pariaconus proboscideus* Percy, 2017	Hawaiian Islands (Hawaii)	KY294095/KY294573 (Percy 2017a)
*Pariaconus pyramidalis* Percy, 2017	Hawaiian Islands (Hawaii)	KY294124/KY294607 (Percy 2017a)
*Pariaconus mauiensis* Percy, 2017	Hawaiian Islands (Maui)	KY293841/KY294316 (Percy 2017a)
*Stevekenia aiea* Percy, 2017	Hawaiian Islands (Kauai)	KY971542/KY971544 (Percy 2017b)
*Stevekenia nothocestri* Percy, 2017	Hawaiian Islands (Oahu)	KY971541/KY971543 (Percy 2017b)
*Trioza remota* Foerster, 1848	England	KY294162/KY294646 (Percy 2017a)
*Trioza urticae* (Linné, 1758)	England	KY011191/KY011286 (Wonglersak et al. 2017)
*T. urticae*	Greece	KY011122/KY011219 (Wonglersak et al. 2017)
	Norway	KY011175/KY011270 (Wonglersak et al. 2017)
	Poland	KY011114/KY011212 (Wonglersak et al. 2017)

Abbreviations used in the descriptions and given in Tables 2–4 are as follows (all measurements are recorded in mm). Adults: WL, fore wing length; WW, fore wing width; HW, head width; VW, vertex width; AL, antennal length; PB, distal proboscis segment length; WL:WW, ratio fore wing length:width; WL:Rs, ratio fore wing length:vein Rs length; CUR, ratio fore wing cell cu_1_ width:height; MR, ratio fore wing cell m_2_ width:height; HW:VW, ratio head width:vertex width; VL:GP, ratio vertex length:genal process length; VL:VW, ratio vertex length:width; AL:HW ratio antennal length:head width; HW:HT ratio head width:hind tibia length; HT:HF, ratio hind tibia length:femur length. Adult male terminalia: MP, proctiger length; PL, paramere length; AEL, distal aedeagus segment length; PL:HW, ratio paramere length:head width; MP:PL, ratio proctiger length:paramere length; PL:AEL, ratio paramere length:distal aedeagus segment length; AEL:AELH, ratio distal aedeagus segment length:aedeagus apical head length; PL:SH, ratio paramere length:subgenital plate height. Adult female terminalia: FP, proctiger length; FSP, subgenital plate length; RL, anal ring length; EL, egg length; FP:HW, ratio female proctiger:head width; FP:RL, ratio female proctiger:anal ring length; FP:SP: ratio female proctiger:subgenital plate length. Immatures: BL, body length; BW, body width; WPL, fore wing pad length; CPL, caudal plate length; CPW, caudal plate width; RW, circumanal ring width; HW, head width; AL, antennal length; BL:BW ratio body length:width; HW:AL ratio head width:antennal length; CPW:RW ratio caudal plate width:circumanal ring width.

**Table 2.** Adult *Swezeyana* measurements (mm).

Group	Species	n	WL	WW	HW	VW	AL	GP	PB	MP	PL	AEL	FP	FSP	RL	EL
***elongagena***	*elongagena*	4m 3f	2.33–2.82	0.67–0.80	0.55–0.60	0.32–0.36	0.82–0.97	0.41–0.48	0.08	0.05–0.07	0.15–0.17	0.10–0.11	0.33–0.34	0.23–0.26	0.17–0.23	0.18–0.20
	*atra*	2m 2f	1.91–2.58	0.55–0.71	0.50–0.55	0.27–0.32	0.64–0.65	0.32–0.35	0.05–0.06	0.05–0.06	0.09	0.08–0.10	0.26–0.31	0.23–0.24	0.13–0.15	–
	*hawaiiensis*	0m 2f	2.33–2.38	0.73–0.76	0.55–0.58	0.30–0.35	0.55	0.31–0.33	0.06	–	–	–	0.32	0.30	0.18	–
	*magna*	1m 0f	3.38	0.92	0.70	0.39	1.12	0.52	0.08	0.13	0.17	0.15	–	–	–	–
	*oahuensis*	4m 3f	2.09–2.88	0.61–0.85	0.49–0.58	0.28–0.33	0.68–0.88	0.35–0.36	0.06–0.07	0.05	0.12–0.13	0.08–0.10	0.27–0.30	0.20–0.24	0.14–0.16	–
	*rubra*	2m 7f	1.79–2.30	0.61–0.79	0.50–0.57	0.29–0.33	0.52–0.56	0.27–0.33	0.05–0.07	0.10–0.11	0.11	0.10–0.11	0.33–0.39	0.23–0.28	0.17–0.21	0.16–0.21
***reticulata***	*reticulata*	5m 5f	1.91–2.58	0.55–0.79	0.45–0.53	0.26–0.30	0.61–0.71	0.30–0.38	0.06	0.09–0.10	0.09–0.10	0.11–0.14	0.34–0.43	0.22–0.30	0.14–0.19	0.12–0.21
	*tentaculata*	4m 4f	1.94–2.53	0.58–0.77	0.47–0.55	0.27–0.32	0.76–0.77	0.35–0.44	0.07–0.08	0.10–0.12	0.11–0.12	0.13–0.14	0.46–0.52	0.36–0.38	0.18–0.23	0.20
**unplaced**	*magnaccai*	4m 0f	1.55–1.76	0.45–0.52	0.41–0.42	0.24	0.58	0.26–0.32	0.05–0.06	0.08–0.10	0.10	0.10–0.13	–	–	–	–

**Table 3.** Adult *Swezeyana* ratios.

Group	Species	WL:WW	CUR	MR	HW:VW	HW:GP	VL:GP	VL:VW	AL:HW	HW:HT	HT:HF
***elongagena***	*elongagena*	3.42–3.66	1.71–2.06	0.75–0.90	1.64–1.71	1.20–1.32	0.54–0.63	0.74–0.82	1.54–1.78	1.63–1.65	0.79–0.92
	*atra*	3.40–3.68	1.59–1.82	0.78–0.86	1.70–1.83	1.43–1.64	0.57–0.73	0.72–0.76	1.30	1.79–1.94	0.77–0.83
	*hawaiiensis*	3.14–3.21	1.35–1.61	0.75–0.83	1.65–1.80	1.73–1.76	0.78–0.82	0.78–0.80	0.95–1.00	2.00–2.11	0.75–0.82
	*magna*	3.66	2.18	0.85	1.77	1.35	0.59	0.77	1.61	1.64	0.85
	*oahuensis*	3.36–3.57	1.63–2.08	0.78–0.93	1.68–1.79	1.40–1.58	0.64–0.75	0.77–0.84	1.39–1.53	1.74–1.90	0.72–0.86
	*rubra*	2.88–3.06	1.15–1.32	0.77–0.92	1.65–1.76	1.69–1.89	0.69–0.87	0.68–0.75	0.93–1.03	1.82–2.09	0.75–0.87
***reticulata***	*reticulata*	3.27–3.55	1.50–1.71	0.58–0.93	1.65–1.78	1.39–1.60	0.60–0.76	0.75–0.82	1.25–1.40	1.53–1.88	0.74–0.83
	*tentaculata*	3.16–3.39	1.33–1.64	0.83–1.00	1.67–1.72	1.15–1.35	0.56–0.63	0.76–0.83	1.40–1.61	1.41–1.80	0.71–0.86
**unplaced**	*magnaccai*	3.29–3.50	1.58–1.73	0.80–0.90	1.69–1.73	1.31–1.59	0.62–0.76	0.81	1.41	1.73–2.25	0.63–0.76

**Table 4.** Adult *Swezeyana* ratios for male and female terminalia.

Group	Species	PL:HW	MP:PL	PL:AEL	AEL:AELH	PL:SH	FP:HW	FP:RL	FP:SP
***elongagena***	*elongagena*	0.29–0.31	0.31–0.43	1.46–1.56	2.00–2.33	0.95–1.05	0.55–0.56	1.41–1.95	1.24–1.45
	*atra*	0.18	0.59–0.73	0.92–1.10	2.00–2.18	0.67–0.85	0.51–0.57	2.05–2.06	1.10–1.34
	*hawaiiensis*	–	–	–	–	–	0.59	1.82	1.05
	*magna*	0.24	0.76	1.14	2.64	1.05	–	–	–
	*oahuensis*	0.25–0.26	0.38	1.19–1.60	2.00–2.17	0.94–1.00	0.53	1.90–2.05	1.27–1.36
	*rubra*	0.22	0.96–1.00	1.04–1.08	2.25–2.27	0.80	0.61–0.69	1.71–2.19	1.20–1.41
***reticulata***	*reticulata*	0.19	1.00–1.18	0.65–0.79	2.27–2.80	0.79–0.92	0.69–0.78	2.17–2.45	1.34–1.56
	*tentaculata*	0.25–0.26	0.93–1.03	0.81–0.88	2.25–2.57	0.78–0.93	0.86–0.95	2.24–2.59	1.27–1.42
**unplaced**	*magnaccai*	0.24–0.25	0.76–0.92	0.78–1.08	2.18–2.67	0.83–1.00	–	–	–

## Taxonomic treatment

### Triozidae Löw, 1879

#### Swezeyana

Taxon classificationAnimaliaHemipteraTriozidae

Caldwell, 1940

Swezeyana Caldwell, 1940: 389. Type species: Swezeyana elongagena Caldwell, 1940, by original designation.

##### Description.

**Adult.** General colour variable ranging from pale yellow-brown, to green or yellow-green, to almost black; often with pink or reddish highlights on the fore wing as well as on the body, especially genal processes, legs, and abdomen. Fore wing membrane either with distinct darker patches or clouds of pigmentation, these range from dark brown to red, and in some cases are limited to termination of veins at wing margins and around cross veins between Rs and wing margin, if without distinct patterns of pigmentation, appearing uniformly clear, opaque yellow or fuscous; wing veins pale to red or dark brown, cross veins between Rs and ventral wing margin with or without pigmentation. Adult length including fore wing from 2–5 mm. Fore wing elongate and usually narrow (ratio WL:WW > 2.80, often > 3), acute to bluntly acute apically, either with trifurcation of veins R, M and Cu_1_, or with vein R branching anterior of bifurcation of M and Cu_1_; vein Rs long, reaching wing margin distad of M fork, but either with or without complete extension of Rs to wing margin, incomplete termination of Rs usually marked by pigmentation; vein R shorter than Cu_1_ and terminating at base of Rs, a pseudopterostigma is present between base of Rs and wing margin, and a more or less thickened wing margin (C+Sc) is present from the wing base to the pseudopterostigma, in some cases occupying part or entire area of cell c+sc; with or without one or more partial or complete cross veins traversing cell r_1_ between vein Rs and ventral wing margin; a single, broadly shaped marginal cluster of radular spines (Figs 3O, 6J) in cells cu_1_, m_1_, and m_2_; surface spinules either present in all cells, dense or sparsely distributed, or few to absent from c+sc, r_2_ and r_1_, often relatively sparsely distributed but becoming denser towards wing margin. Hind wing narrow and elongate (> 0.5 length of fore wing), clear or slightly fuscous in basal half. Head moderately deflexed downwards, vertex more or less flat dorsally, with lateral ocelli lying on small tubercles, medial epicranial suture distinct; genal processes extremely long, often upturned at apices, with scattered long setae and usually a single, long subapical seta on each process. Antennae short; antennal segments 10, either entirely dark, or more usually with terminal 3(-7) segments darker, or distal part of segments 3-8 darker; a single rhinarium apically on each of segments 4, 6, 8, 9; 1-2 long setae on each of segments 3-9, terminal segment with two unequal length apical setae. Distal proboscis segment short, darker apically. Thorax somewhat flattened to only moderately arched; vertex and thorax with scattered short to moderately long setae. Legs short, hind legs robust with femur longer than tibia; hind leg with meracanthus reduced to almost absent; metafemur with several stout setae apically; metatibia with or without distinct genual spine basally and typically with 1+2 (occasionally 1+3) sclerotized apical spurs; pro- and mesotarsi subequal in length, metatarsi unequal with extremely long basal tarsus slightly expanded with concave, ridged underside (Fig. 3M). Male terminalia with somewhat elongate subgenital plate; proctiger with pronounced posterior lobes medially, 1-2 long setae usually present on posterior apices of each lobe, length shorter, subequal or longer than paramere; paramere shape variable, generally broad basally and tapering to apex, with two stout setae on the interior apex (sometimes appearing as one from lateral view); distal aedeagus segment apex hooked. Female terminalia with medium to long dorsal and ventral setae; proctiger either truncate and markedly convex apically, with apex broad, blunt, bearing small medial cleft and fringed with stout setae, or dorsal surface more or less straight, apex tapering, lacking medial cleft and distinct fringe of setae; proctiger longer than subgenital plate; anal ring hour-glass shaped (with or without a head compartment at proximal end) and composed of a, usually, uninterrupted, double row of cells, posterior/distal portion of ring margin either smooth or convoluted; subgenital plate ventral surface either convex or more or less straight, apex terminating in a variably shaped beak often bearing a short or more pronounced medial cleft spanned by a short or extended membrane; ovipositor valves small, without serrations (Fig. 9O).

**Egg.** Known for four species. Pale or light brown, oblong-ovoid with a short, laterally positioned pedicel sub-basally on underside; distinctly hexagonal, honeycomb-like, sculpturing, to semi-hexagonal or rounded indentations dorsally; underside unsculptured, tail apparently lacking.

**Immature.** Known for four species. 5^th^ instar oblong-ovoid, ventro-dorsally flattened with slightly protruding wing buds and distinct humeral lobes; antennae with 3(-4) segments bearing 3(-4) rhinaria (1 on segments 2-3, and 2 on apical segment) and two long, terminal, simple setae of unequal length; tarsi with broad crescent arolia and extremely small, reduced claws; each terminal tarsus bearing a long capitate seta; anus situated ventrally, circumanal ring broad and composed of a single row of elongate cells; dorsum either with wax producing pores (see Tuthill 1966), or non-wax producing tubercles and tentacles (Fig. 12). Chaetotaxy: 5^th^ instar with either continuous or interrupted coverage of marginal setae; overall setal types, even between closely related species (e.g., *S. reticulata* and *S. tentaculata*), highly variable (Fig. 12). Smaller instars only known for *S. reticulata* and *S. tentaculata*, in which tubercles are apparent from 2^nd^ instar (Fig. 13).

##### Biology.

All species for which the biology is known have free-living immatures on the surface of leaves (either lower, or both upper and lower surfaces). Those species with immatures described here with protruding tubercles and tentacles were mostly found on the lower leaf surface among dense indumentum and often close to the mid-rib (Fig. 12N).

##### Host plant.

All *Swezeyana* are host specific on a single Hawaiian endemic host plant species, *Planchonella sandwicensis* (Sapotaceae).

##### Comments.

Two species groups are recognized, *elongagena* group and *reticulata* group, based primarily on the strikingly different forms of female terminalia. The *elongagena* group has broad, truncate female terminalia with a strongly convex proctiger apex; the proctiger and subgenital plate bear a small to pronounced medial cleft at the apex. In contrast, the *reticulata* group has tapering terminalia without a medial cleft in the proctiger apex. In both species groups the subgenital plate terminates in a more or less well developed beak with small to pronounced cleft spanned by a membrane. The underlying endoskeleton of the two different forms of female terminalia indicate distinctly different development of the apodemes in the two species groups: broad and short in *elongagena* group (Fig. 4P), and long and narrow in *reticulata* group (Fig. 10I). However, not all species treated here are known for both sexes, therefore current assignment to these groups relies on other characteristics and DNA barcode data (see Discussion). Due to the unknown female morphology and inconclusive placement in the molecular phylogeny, *S. magnaccai* is not placed within a species group. Fore wing characteristics such as wing membrane colouration and pseudoveins/cross veins are found in both groups. The 5^th^ instar immatures may also be diagnostic, with *elongagena* group having wax producing pores dorsally (illustrated in Tuthill 1966 for *S. elongagena*) and continuous ring of marginal setae (illustrated in Caldwell 1940 for *S. elongagena*); while in contrast, the *reticulata* group are characterized by non-wax producing tubercles and tentacles and lack a contiguous marginal ring of setae (Figs 12–13). However, currently immatures are known for these three species only, so it remains to be tested whether these highly distinct immature morphologies reflect species group assignments.

##### Note on adult assignment to species group.

The two species groups (*elongagena* group and *reticulata* group) are most easily recognized by the shape of the female terminalia, e.g., extremely convex apex of female proctiger in the *elongagena* group, versus more or less dorsally straight and tapering in the *reticulata* group. The *elongagena* group females have a FP:HW ratio typically < 0.70 (range 0.51–0.69), and FP:RL ratio typically < 2.18 (range 1.41–2.19); whereas in the *reticulata* group FP:HW ratio is typically > 0.70 (range 0.69–0.95), and FP:RL ratio is typically > 2.18 (range 2.17–2.59). *Swezeyana* males are less easily assigned to a species group, but *elongagena* group males have a distal aedeagus segment that is typically shorter than the paramere (PL:AEL ratio range 0.92–1.60), whereas *reticulata* group males have a distal aedeagus segment that is longer than the paramere (PL:AEL ratio range 0.65–0.88). Notably, the fore wing characters used by Zimmerman (1948) to key out the two species described by Caldwell (*S. elongagena* and *S. reticulata*), such as presence/absence of cross veins in cell r_1_ and presence/absence of distinct patterns of pigmentation are found in both species groups. The key below does not key to species group, rather it employs characters useful in distinguishing species, in particular those co-occurring on the same island.

##### Note on molecular analyses.

The neighbour-joining analysis of two mitochondrial DNA regions is presented in Fig. 2. Strong support is recovered for the *reticulata* group, but not for the *elongagena* group. The topologies recovered in comparative ML and Bayesian analyses differ only at weakly or unsupported nodes. The three nodes that group same island sister taxon pairs (on Kauai, Oahu, and Hawaii) are recovered in all analyses but with variable support (much stronger support in the Bayesian than NJ or ML analyses) (see Fig. 2 and Discussion). Maximum genetic divergence (uncorrected p-distances) among *Swezeyana* species is 19.9%; maximum intraspecific divergence (3%) was found in *S. magnaccai* on Oahu.

##### Key to Swezeyana adults

**Table:** 

1	Fore wings with distinct darker patches or clouds of pigmentation, in some cases only around termination of veins at wing margins and around cross veins between Rs and wing margin in cell r_1_ (Fig. 1E–I)	**2**
–	Fore wings without distinct darker patches or clouds of pigmentation, appearing uniformly clear or opaque, cross veins between Rs and wing margin in cell r_1_, if present, unpigmented (Fig. 1A–D)	**6**
2	Antennae short, subequal to head width (ratio AL:HW < 1.1), ratio HW:GP > 1.65, female proctiger strongly convex apically with apex extremely broad and blunt, ratio FP:HW < 0.70, on Hawaii	**3**
–	Antennae longer (AL:HW > 1.1), ratio HW:GP < 1.65, female proctiger more or less straight dorsally with apex bluntly acute, on other islands	**4**
3	Fore wings with extensive patches and clouds of red-brown pigmentation, particularly across the central area of wing, and numerous cross veins (typically more than 5) between Rs and wing margin in cell r_1_, smaller species with broader wings (ratio WL:WW < 3.1) and wing cell cu_1_ relatively narrow and high (ratio CUR < 1.33) (Figs 1I, 8A–W)	***S. rubra* sp. n.**
–	Fore wings with only indistinct fuscous brown on membrane and distinct small darker brown patches around termination of veins at wing margins and around cross veins between Rs and wing margin in cell r_1_, cross veins fewer (typically less than 5), larger species with narrower wings (ratio WL:WW > 3.1) and wing cell cu_1_ relatively wide and low (ratio CUR > 1.33) (Figs 1E, 5I–K)	***S. hawaiiensis* sp. n.**
4	Larger species (WL > 1.9 mm), paramere shape more triangular with a broader base, on Kauai (possibly other islands for *S. reticulata*)	**5**
–	Smaller species (WL < 1.9 mm), paramere shape more slender with a narrower base, on Oahu (Figs 1F, 11)	***S. magnaccai* sp. n.**
5	Fore wing with fewer cross veins (typically less than 6) between Rs and wing margin, head with shorter genal processes (ratio HW:GP > 1.36) and shorter antennae (AL < 0.75 mm, ratio AL:HW ≤ 1.40), paramere shorter and broader (ratio PL:HW < 0.20, ratio PL:AEL < 0.80), female terminalia shorter (ratio FP:HW < 0.80) with posterior margin of anal ring less convoluted and incised (Figs 1G, 9A–T; immatures Figs 12A–G, 13A–F)	***S. reticulata* Caldwell, 1940**
–	Fore wing with more cross veins (typically more than 6) between Rs and wing margin, head with longer genal processes (ratio HW:GP < 1.36) and longer antennae (AL > 0.75 mm, ratio AL:HW ≥ 1.40), paramere longer and narrower (ratio PL:HW > 0.20, ratio PL:AEL > 0.80), female terminalia longer (ratio FP:HW > 0.80) with posterior margin of anal ring more convoluted and incised (Figs 1H, 10A–S; immatures Figs 12H–N, 13G–J)	***S. tentaculata* sp. n.**
6	Smaller species (WL < 3 mm, AL < 1 mm), wing cell cu_1_ relatively narrow and high (ratio CUR < 2.1), on Oahu, Molokai, Maui	**7**
–	Larger species (WL > 3 mm, AL > 1 mm), wing cell cu_1_ relatively wide and low (ratio CUR > 2.1), on Kauai (Figs 1D, 6A–K)	***S. magna* sp. n.**
7	Genal processes shorter (GP < 0.40 mm, ratio HW:GP > 1.35), paramere shorter (ratio PL:HW < 0.27) and with less extended anteriorly directed apex, on Oahu	**8**
–	Genal processes longer (GP > 0.40 mm, ratio HW:GP < 1.35), paramere longer (ratio PL:HW > 0.27) and with more extended anteriorly directed apex, on Molokai and Maui (Figs 1A, 3A–P)	***S. elongagena* Caldwell, 1940**
8	Paler species (generally yellow-brown to green), fore wing vein Rs typically longer relative to wing length (ratio WL:Rs 1.57–1.82) resulting in shorter distance between terminations of Rs and M_1+2_, antennae longer (AL > 0.65 mm), paramere longer (ratios PL:HW > 0.20, PL:AEL > 1.15, PL:SH > 0.90) (Figs 1B, 7A–R)	***S. oahuensis* sp. n.**
–	Darker species (generally dark brown to black), fore wing vein Rs typically shorter relative to wing length (ratio WL:Rs 1.76–2.03) resulting in longer distance between terminations of Rs and M_1+2_, antennae shorter (AL ≤ 0.65 mm), paramere shorter (ratios PL:HW < 0.20, PL:AEL < 1.15, PL:SH < 0.90) (Figs 1C, 4A–T)	***S. atra* sp. n.**

##### Note on species descriptions.

*Swezeyana* is a small genus with, in general, considerable morphological homogeneity. The species descriptions below provide details of species specific characteristics not supplied in the generic description above.

#### Species group: *elongagena*

##### Swezeyana elongagena

Taxon classificationAnimaliaHemipteraTriozidae

Caldwell, 1940

Figures 1A , 3

Swezeyana elongagena Caldwell, 1940: 390.

###### Description.

**Figure 1.**
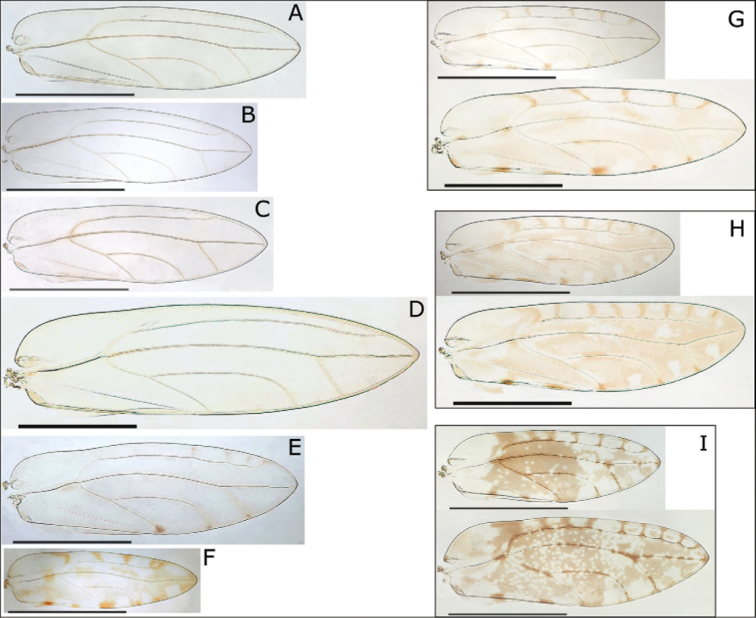
Fore wings of nine *Swezeyana* species: **A** *S. elongagena* (male) **B** *S. oahuensis* (male) **C** *S. atra* (male) **D** *S. magna* (male) **E** *S. hawaiiensis* (female) **F** *S. magnaccai* (male) **G** *S. reticulata* (male above, female below) **H** *S. tentaculata* (male above, female below) **I** *S. rubra* (male above, female below). Scale bars: 1 mm.

**Adult.** General body colour green to yellow-green or yellow-brown, last 3-5 antennal segments darker brown to black, apices of genae sometimes pinkish-red. Fore wing membrane uniformly pale fuscous (Fig. 1A). Fore wing apex acute to bluntly acute; pseudopterostigma relatively short to medium long (Fig. 3P), none to few (1-3) cross pseudoveins in cell r_1_; surface spinules sparsely distributed, apparently absent from c+sc (Fig. 3O); medium long setae on ventral margin and medium short to short setae on veins and dorsal margin. Antennae medium long (ratio AL:HW 1.54–1.78) (Fig. 3D); genal processes long (GP > 0.40 mm, ratio HW:GP < 1.35) and often upturned at apices (Fig. 3A–B); short to medium short setae on vertex and thorax. Meracanthus small (Fig. 3N), genual spine reduced (Fig. 3L). Male terminalia (Fig. 3G–I): paramere slender, long (ratio PL:HW > 0.27), tapering to anteriorly projecting apex with two short stout setae; distal aedeagus segment short relative to paramere (ratio PL:AEL 1.46–1.56), apex developed into a large rounded hook with blunt apex. Female terminalia (Fig. 3E–F): proctiger dorsal surface strongly convex apically, apex broad, blunt, bearing medial cleft and fringed with stout setae, anal ring long (ratio FP:RL 1.41–1.95), with reduced head compartment at proximal end, distal portion of ring margin smooth; subgenital plate convex with medial cleft pronounced (almost half length of subgenital plate).

**Egg.** Pale with well-defined hexagonal (honeycomb-like) sculpturing dorsally (Fig. 3J).

**Immature.** Described and illustrated by Caldwell (1940) and Tuthill (1966). Although Caldwell refers to marginal “sectasetae” in his description, he illustrates distinctly fan-shaped and apparently unbisected setae. Tuthill provides an image of an immature with long “wax filaments” produced by dorsal pores, and notes that free-living immatures were observed mostly on the upper, but also lower, leaf surfaces. Both descriptions suggest notable morphological differences to immatures described here for *S. reticulata* and *S. tentaculata* sp. n.

###### Host plant.

*Planchonella sandwicensis*.

###### Distribution.

Maui, Molokai (a single female specimen recorded from Kauai, and apparently now missing, is queried in Zimmerman (1948), and may have been *S. magna* sp. n.). Specimens from Molokai are close to the type specimens from Maui, and currently this species is considered to be restricted to these two islands.

###### Comments.

Although Caldwell’s (1940) illustration only indicates a single cross pseudovein, examination of the type specimen revealed three unpigmented pseudoveins. A single female paratype collected from Kalalau Trail, Kauai, is likely to be *S. magna* sp. n. (see also assignment in error comment in Zimmerman 1948)

###### Material examined.

Holotype female (slide mounted), Haelaau, Maui, USA, ex *Planchonella* sp., 19 December 1928 (BPBM). Paratypes: 10m 5f, same data as holotype (not located). Other material: 5m 4f, Kamakou Preserve, Molokai, USA, N21.1236, W-156.9108, ex *Planchonella sandwicensis*, 17 August 2003, “Hi20-03” D. Percy leg. (BMNH).

###### Gene sequences.

MG988832 (COI) MG989153 (cytB) (Hi20-03).

##### Swezeyana atra sp. n.

Taxon classificationAnimaliaHemipteraTriozidae

http://zoobank.org/3BA915C9-4A1D-4910-A5A7-F7F130BF1314

Figures 1C , 4

###### Diagnosis.

Medium sized, dark coloured species, with fore wing membrane unpatterned, antennae medium short, genal processes long, paramere short, and female proctiger strongly convex apically.

###### Description.

**Adult.** General body colour brown to black, particularly dark on the dorsum of head and thorax. Fore wing membrane generally uniformly clear or slightly fuscous, with fuscous-brown cloud usually present around vein R and basal dorsal claval margin (Fig. 1C). Fore wing apex acute; pseudopterostigma relatively long (Fig. 4S), typically no cross pseudovein in cell r_1_ (one individual seen with partial cross vein); surface spinules relatively sparsely distributed but denser towards wing margin, few or absent from c+sc; medium long setae on ventral margin and medium short to short setae on veins and dorsal margin. Antennae medium short (ratio AL:HW 1.30) (Fig. 4B–C); genal processes medium long (GP < 0.40 mm, ratio HW:GP > 1.40) and slightly upturned at apices (Fig. 4B); medium short to short setae on vertex and thorax. Meracanthus extremely small (Fig. 4Q), genual spine reduced or absent (Fig. 4R). Male terminalia (Fig. 4F–I): paramere short (ratio PL:HW < 0.20), tapering to slightly anteriorly projecting apex with two short stout setae; distal aedeagus segment long relative to paramere (ratio PL:AEL 0.92–1.10), apex developed into a large rounded hook with blunt apex. Female terminalia (Fig. 4J–P): proctiger dorsal surface strongly convex apically, apex broad, blunt, bearing medial cleft and fringed with stout setae, anal ring medium long (ratio FP:RL > 2.00), without or with reduced head compartment at proximal end, distal portion of ring margin smooth; subgenital plate slightly convex with short medial cleft (Fig. 4N–O).

**Egg.** Unknown.

**Immature.** Unknown.

###### Host plant.

*Planchonella sandwicensis*.

###### Distribution.

Oahu. Only known from the Waianae Mountains.

###### Etymology.

Named for the dark body colouration, especially the head and thorax (adjective in the nominative singular).

###### Comments.

**Figure 2.**
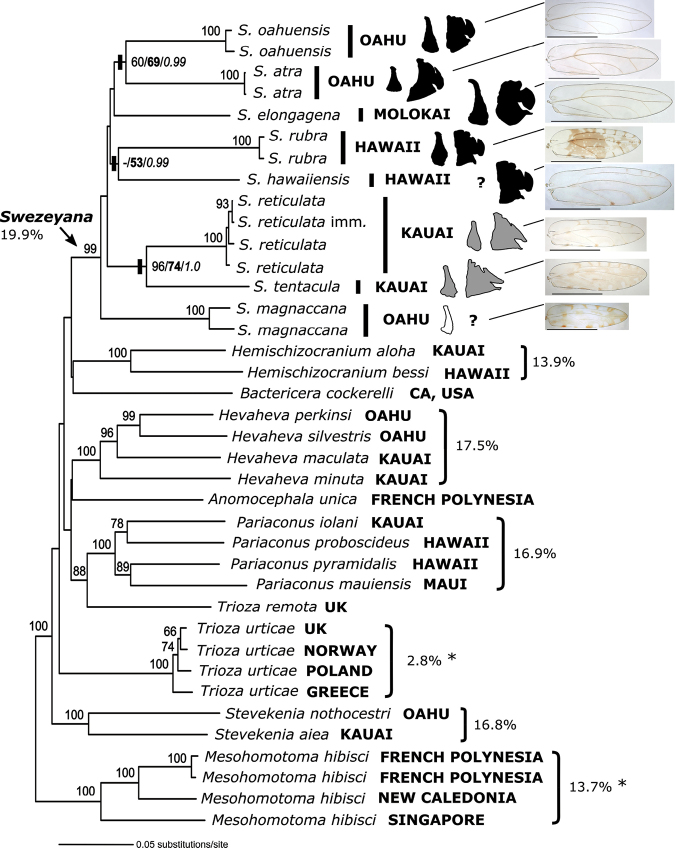
Neighbour-joining (NJ) analysis (combined COI and cytB data) with 1000 bootstrap replicates in PAUP*. Eight of the nine *Swezeyana* species are included and 16 other taxa from Triozidae, mostly representing other Hawaiian genera for comparison of divergence; and one species from Carsidaridae as an outgroup (*Mesohomotoma hibisci*) (Table 1). Outlines of parameres and female terminalia illustrate the two recognized species groups (*elongagena* group in black, *reticulata* group in grey, *S. magnaccai* unplaced), and fore wing images (Fig. 1) show the distribution of patterned/pigmented wings. Regional localities of sample are given, and “imm.” indicates an immature sampled for *S. reticulata*. NJ bootstrap support values ≥ 60% are indicated for all nodes, and comparative node support from ML and Bayesian analyses are shown for the three same island sister taxa pairs (NJ/ML[bold]/Bayesian[italic]) (see Discussion). Maximum genetic divergence (uncorrected p-distances) is shown for *Swezeyana* and four other endemic Hawaiian genera; also shown are maximum intraspecific distances (indicated with an asterix) for two widespread non-Hawaiian species with multiple individuals sampled across terrestrial (*T. urticae*) or oceanic (*M. hibisci*) landscapes (see Discussion).

This is the darkest of the *Swezeyana* species. It has the shortest paramere in the *elongagena* group, with the shape more similar to those in the *reticulata* group. The distribution in the Waianae Mountains is shared with *S. oahuensis*, and the molecular topology places these two species as sister taxa but with weak bootstrap support (Fig. 2).

**Figure 3.**
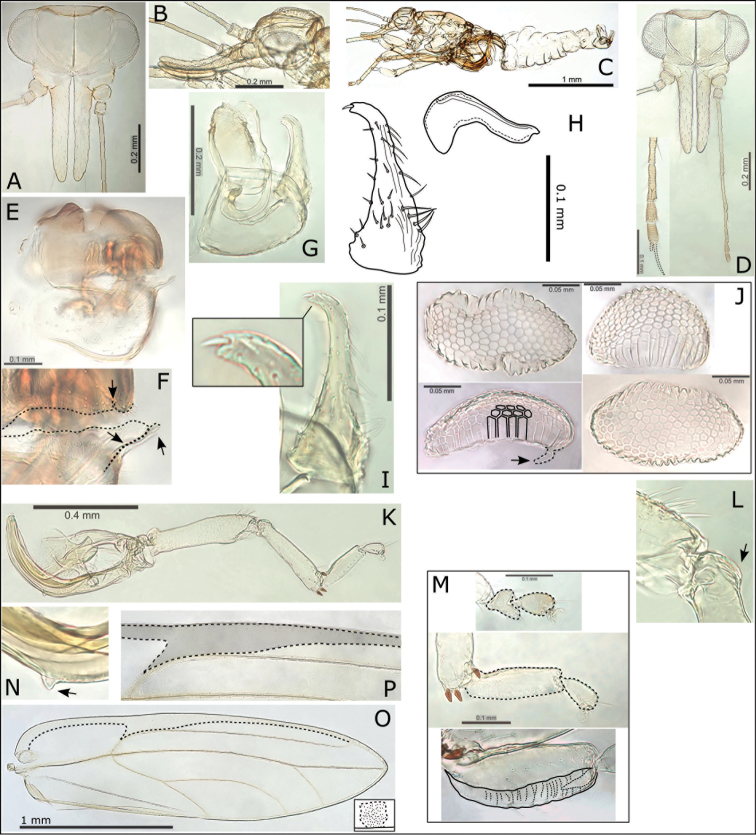
*Swezeyana elongagena*. **A** head **B** head (lateral view) **C** male **D** head and antenna, inset detail of terminal antennal segments (terminal setae outlined) **E** female terminalia **F** detail of apex of female terminalia indicating beak and position of medial clefts in proctiger and subgenital plate (outlined) **G** male terminalia **H** paramere and distal aedeagus segment **I** paramere, inset detail of apex **J** eggs (distinctly hexagonal sculpturing and pedicel outlined and indicated) **K** hind leg **L** base of hind tibia with reduced genual spine indicated **M** mesotarsi and metatarsi (outlined), concave and ridged underside of basal metatarsus (outlined) **N** small but distinct meracanthus (indicated) **O** fore wing with interior edge of ventral margin outlined, inset illustrating broad shape of marginal radular spine cluster **P** extent of fore wing pseudopterostigma (shaded).

**Figure 4.**
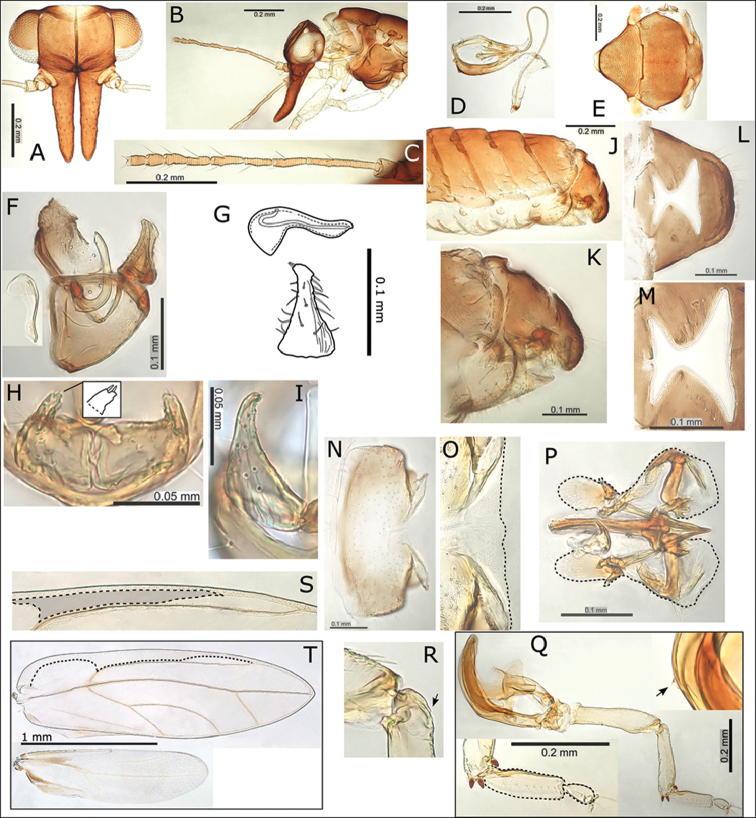
*Swezeyana atra* sp. n. **A** head **B** head and antenna (lateral view) **C** antenna **D** proboscis **E** dorsum of thorax **F** male terminalia, inset distal aedeagus segment **G** paramere and distal aedeagus segment **H** parameres (dorsal view), inset illustrating paramere apex **I** paramere (posterior view) **J** female abdomen **K** female terminalia **L** female proctiger (dorsal view) **M** detail of anal ring **N** female subgenital plate (ventral view) **O** detail of posterior apex of female subgenital plate with membrane (outlined) **P** female terminalia endoskeleton (dorsal view, outlined) **Q** hind leg, inset reduced meracanthus (indicated) and metatarsi (outlined) **R** base of hind tibia with highly reduced genual spine (indicated) **S** extent of fore wing pseudopterostigma (shaded) **T** fore wing (above) with interior edge of ventral margin outlined, hind wing (below).

###### Material examined.

Holotype male (slide mounted), Waianae Mnts, Oahu, USA, N21.4585, W-158.0973, ex *Planchonella sandwicensis*, 5 July 2014, “Hi65-14” D. Percy leg. (BMNH). Paratype (slide mounted) 1f, as for holotype (BMNH). Other material: 1m 1f, Puu Hapapa, Central Waianae Mnts, Oahu, USA, N21.4666, W-158.1029, ex *Planchonella sandwicensis*, 17 May 2014, “KM14-14” K. Magnacca leg. (BMNH).

###### Gene sequences.

MH001521 (COI) MH001527 (cytB) (Hi65-14); MH001522 (COI) MH001528 (cytB) (KM14-14).

##### Swezeyana hawaiiensis sp. n.

Taxon classificationAnimaliaHemipteraTriozidae

http://zoobank.org/68663D7C-532C-4158-9A58-FF189893B6D3

Figures 1E , 5

###### Diagnosis.

Medium sized, light coloured species, with fore wing membrane unpatterned, antennae and genal processes relatively short, and female proctiger strongly convex apically.

###### Description.

**Figure 5.**
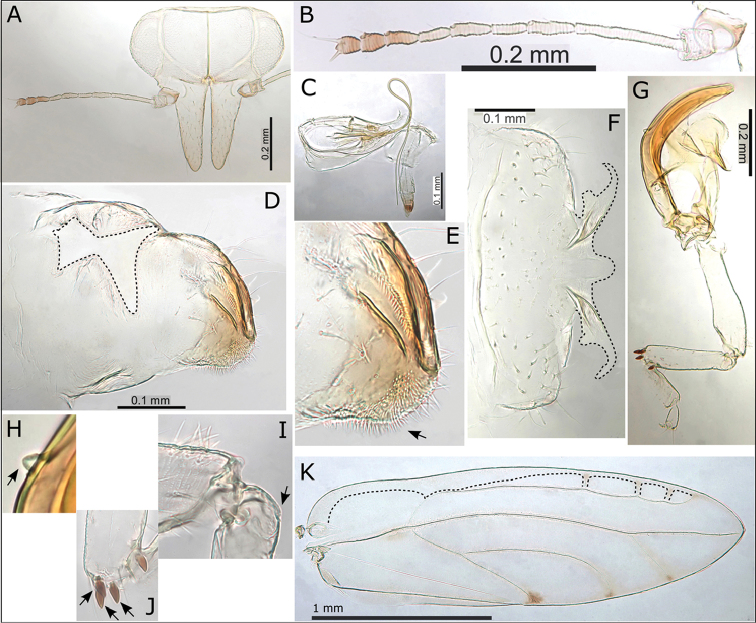
*Swezeyana hawaiiensis* sp. n. (female). **A** head and antenna **B** antenna **C** proboscis **D** proctiger, dorso-lateral view showing anal ring (outlined) **E** detail of apex of proctiger showing medial cleft and fringe of apical setae (indicated) **F** female subgenital plate (ventral view) with apical beak and membrane outlined **G** hind leg **H** small but distinct meracanthus (indicated) **I** base of hind tibia (highly reduced genual spine indicated) **J** detail of atypical 1+3 (indicated) arrangement of sclerotized apical metatibia spurs **K** fore wing, with interior edge of ventral margin outlined.

**Adult.** General body colour green to yellow-green. Fore wing membrane generally slightly fuscous, darker fuscous clouds around cross pseudoveins towards apex of cell r_1_, and distinct brown patches at termination of veins Cu_1a_, Cu_1b_, and M_3+4_ (Figs 1E, 5K). Fore wing apex acute to bluntly acute; pseudopterostigma short (Fig. 5K), 2-3 cross pseudoveins present in apical portion of cell r_1_; surface spinules sparsely distributed and reduced in distribution, few or absent from r_1_, r_2_, and c+sc; medium long setae on ventral margin and medium short to short setae on veins and dorsal margin. Antennae short (ratio AL:HW 0.95–1.00), terminal 3 segments darker brown (Figs 5A–B); genal processes relatively short (GP < 0.35 mm, ratio HW:GP > 1.70), not or only slightly upturned at apices (Fig. 5A); short to minute setae on vertex and thorax. Meracanthus small (Fig. 5H), genual spine reduced or absent (Fig. 5I). Female terminalia (Fig. 5D–F): proctiger dorsal surface strongly convex apically, apex broad, blunt, bearing medial cleft and fringed with stout setae (Fig. 5E), anal ring long (ratio FP:RL 1.82), with head compartment at proximal end, distal portion of ring margin smooth; subgenital plate slightly convex with short medial cleft with beak and membrane extended (Fig. 5F).

**Egg.** Unknown.

**Immature.** Free-living immatures were observed mostly on the upper, but also on the lower, leaf surfaces. Specimens collected were unfortunately lost during specimen shipping.

###### Host plant.

*Planchonella sandwicensis*.

###### Distribution.

Hawaii. Only known from PuuWaaWaa area.

###### Etymology.

Named for its distribution on the island of Hawaii (adjective in the nominative singular).

###### Comments.

This species was collected from the same individual host tree as *S. rubra*; both species are in the *elongagena* group but are easily separated in the field due to general body colour and a distinctly patterned fore wing in *S. rubra*; both species have comparatively short genal processes as well as the shortest antennae in the genus (subequal to head width). The molecular topology places these as sister taxa, but without bootstrap support. Males with the same collection data as females were unfortunately lost during specimen shipping.

###### Material examined.

Holotype female (slide mounted), PuuWaaWaa, Hawaii, USA, N19.784, W-155.833, 820m, ex *Planchonella sandwicensis*, 29 July 2002, “440A-02” D. Percy leg. (BMNH). Paratype (slide mounted) 1f, as for holotype (BMNH).

###### Gene sequences.

MG988835 (COI) MG989155 (cytB) (440A-02).

##### Swezeyana magna sp. n.

Taxon classificationAnimaliaHemipteraTriozidae

http://zoobank.org/8168F0F6-C89C-4488-B637-4D017FA8154A

Figures 1D , 6

###### Diagnosis.

Large, light coloured species, with fore wing membrane unpatterned, antennae and genal processes long, and paramere short.

###### Description.

**Figure 6.**
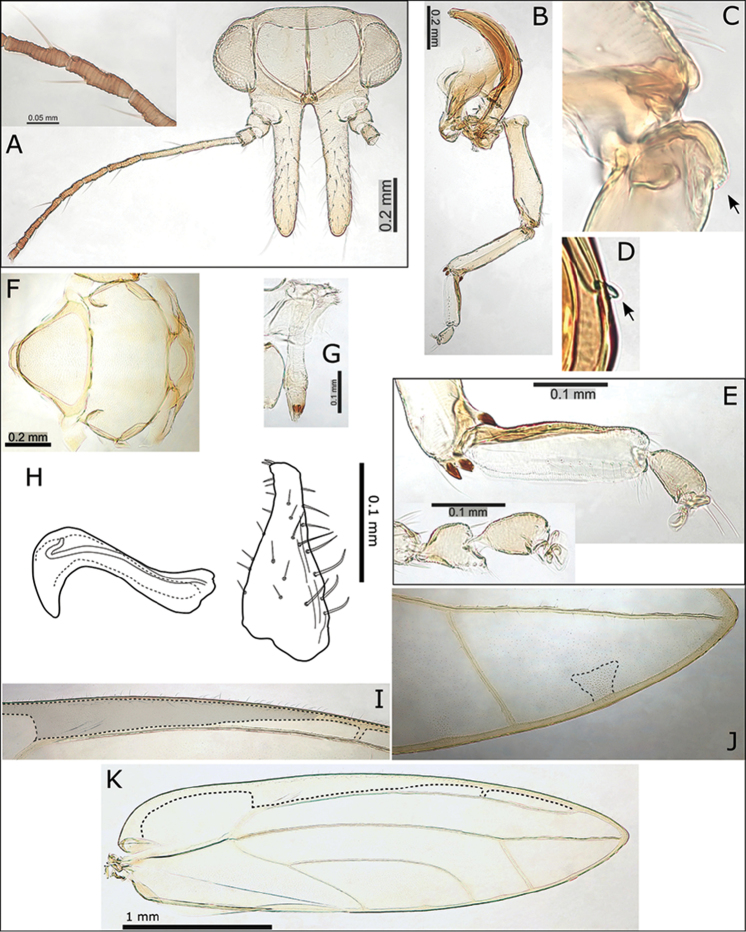
*Swezeyana magna* sp. n. **A** head and antenna, inset antenna detail **B** hind leg **C** base of hind tibia (reduced genual spine indicated) **D** small but distinct meracanthus (indicated) **E** metatarsi, inset comparative size of mesotarsi **F** dorsum of thorax **G** proboscis **H** aedeagus and paramere **I** extent of pseudopterostigma (shaded) **J** broad shape of marginal radular spine cluster (outlined) **K** fore wing, with interior edge of ventral margin outlined.

**Adult.** General body colour yellow-green to yellow-brown, last 7-8 antennal segments darker brown. Fore wing membrane uniformly pale fuscous (Fig. 1D). Fore wing apex acute; pseudopterostigma medium long (Fig. 6I), 0-1 cross pseudoveins in basal portion of cell r_1_; surface spinules sparsely distributed, apparently absent from c+sc; medium long setae on ventral margin and medium short setae on veins and dorsal margin. Antennae long (AL > 1 mm, ratio AL:HW 1.61) (Fig. 6A); genal processes long (GP > 0.50 mm, ratio HW:GP < 1.40) and slightly upturned at apices (Fig. 6A); short to minute setae on vertex and thorax. Meracanthus small (Fig. 6D), genual spine reduced (Fig. 6C). Male terminalia (Fig. 6H): paramere short (ratio PL:HW 0.24), tapering to small, anteriorly projecting apex with two short stout setae; distal aedeagus segment long relative to paramere (ratio PL:AEL 1.14), apex developed into a large rounded hook with bluntly acute apex.

**Egg.** Unknown.

**Immature.** Unknown.

###### Host plant.

*Planchonella sandwicensis*.

###### Distribution.

Kauai. Only known from one location in Kokee State Park.

###### Etymology.

Named for the large body size (adjective in the nominative singular).

###### Comments.

This is the largest *Swezeyana* species and is only known from a single male; fore wing type suggests it is part of the *elongagena* species group, but the paramere shape is somewhat similar to other *reticulata* group species on Kauai. As no molecular sequences are available for this species, the group affiliation remains uncertain, but this species and the other Kauai species may represent early divergence of the *elongagena* and *reticulata* species groups.

###### Material examined.

Holotype male (slide mounted), Kokee State Park, Kauai, USA, N22.1444, W-159.6477, ex *Planchonella sandwicensis*, 29 October 2005, “Hi01-05” D. Percy leg. (BMNH).

##### Swezeyana oahuensis sp. n.

Taxon classificationAnimaliaHemipteraTriozidae

http://zoobank.org/50F9E50B-DA6D-4DD9-BAA5-3B8347B49E16

Figures 1B , 7

###### Diagnosis.

Medium sized, light coloured species, with fore wing membrane unpatterned, antennae medium long, genal processes long, paramere medium long, and female proctiger strongly convex apically.

###### Description.

**Figure 7.**
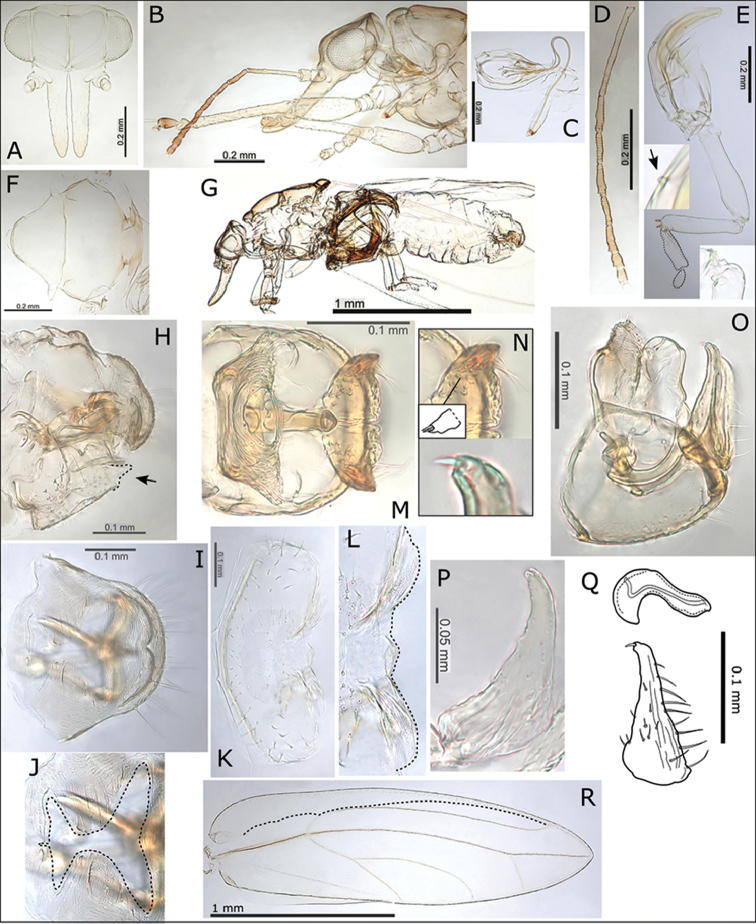
*Swezeyana oahuensis* sp. n. **A** head **B** head and antenna (lateral view) **C** proboscis **D** antenna **E** hind leg, inset detail of reduced meracanthus (indicated) and genual spine **F** dorsum of thorax **G** female **H** female terminalia (truncate subgenital plate indicated, apex outlined) **I** female terminalia (dorsal view) **J** detail of anal ring (outlined) **K** female subgenital plate (ventral view) **L** detail of posterior apex of female subgenital plate with beak and membrane (outlined) **M** male terminalia (dorsal view) **N** paramere apex (dorsal view above and outlined, lateral view below) **O** male terminalia **P** paramere (posterior view) **Q** aedeagus and paramere **R** fore wing, with interior edge of ventral margin outlined.

**Adult.** General body colour green to yellow-green or yellow-brown, last 5-7 antennal segments darker brown, apices of genae sometimes pinkish-red. Fore wing membrane uniformly pale fuscous (Fig. 1B). Fore wing apex acute; pseudopterostigma medium to long (Fig. 7R), no cross pseudoveins in cell r_1_; surface spinules sparsely distributed, reduced or absent from r_1_ and r_2_, and apparently absent from c+sc which is partly to almost entirely composed of a thickened ventral wing margin (C+Sc); medium long setae on ventral margin and medium short to short setae on veins and dorsal margin. Antennae medium long (ratio AL:HW 1.39–1.53) (Fig. 7B, D); genal processes medium long (GP ≥ 0.35 mm, ratio HW:GP ≥ 1.40) and often upturned at apices (Fig. 7A–B, G); short to medium short setae on vertex and thorax. Meracanthus small (Fig. 7E), genual spine reduced or absent (Fig. 7E). Male terminalia (Fig. 7M–Q): paramere slender, medium long (ratio PL:HW ≥ 0.25), tapering to anteriorly projecting apex with two short stout setae; distal aedeagus segment short relative to paramere (ratio PL:AEL 1.19–1.60), apex developed into a large rounded hook with bluntly acute apex. Female terminalia (Figs 7H–L): proctiger dorsal surface strongly convex apically, apex broad, blunt, bearing medial cleft and fringed with stout setae, anal ring medium long (ratio FP:RL 1.90–2.05), with reduced head compartment at proximal end, distal portion of ring margin smooth; subgenital plate slightly convex with short medial cleft with beak and membrane extended (Fig. 7K–L).

**Egg.** Unknown.

**Immature.** Unknown.

###### Host plant.

*Planchonella sandwicensis*.

###### Distribution.

Oahu. Only known from the Waianae Mountains.

###### Etymology.

Named for its distribution on the island of Oahu (adjective in the nominative singular).

###### Comments.

This species and *S. atra* may represent insular diversification on Oahu (see comments for *S. atra*).

###### Material examined.

Holotype male (slide mounted), Mnt Kaala road (culvert 32), Waianae Mnts, Oahu, USA, ex *Planchonella sandwicensis*, 26 August 2003, “Hi57-03” D. Percy leg. (BMNH). Paratypes (slide mounted) 2f, as for holotype (BMNH). Paratypes (slide mounted) 2m, Pahole NAR, Waianaea Mnts, Oahu, USA, N21.5364, W-158.1919, ex *Planchonella sandwicensis*, 14 August 2003, “Hi06-03” D. Percy leg. (BMNH). Other material: 1m 2f, South Mohiakea, Central Waianae Mnts, Oahu, USA, N21.4821, W-158.1247, ex *Planchonella sandwicensis*, 29 January 2014, “KM16-14” K. Magnacca leg. (BMNH).

**Gene sequences.** KY294142 (COI) KY294626 (cytB) (KM16-14) [previously submitted to GenBank as *Swezeyana elongagena* Caldwell, 1940 (in Percy 2017a)]; KY294143 (COI) KY294627 (cytB) (Hi57-03) [previously submitted to GenBank as *Swezeyana elongagena* Caldwell, 1940 (in Percy 2017a)].

##### Swezeyana rubra sp. n.

Taxon classificationAnimaliaHemipteraTriozidae

http://zoobank.org/19F21190-095C-4B30-9CF7-FEF2EFA58EE6

Figures 1I , 8

###### Diagnosis.

Medium small, red-brown species, with fore wing membrane distinctly patterned, antennae and genal processes relatively short, paramere short, and female proctiger strongly convex apically.

###### Description.

**Figure 8.**
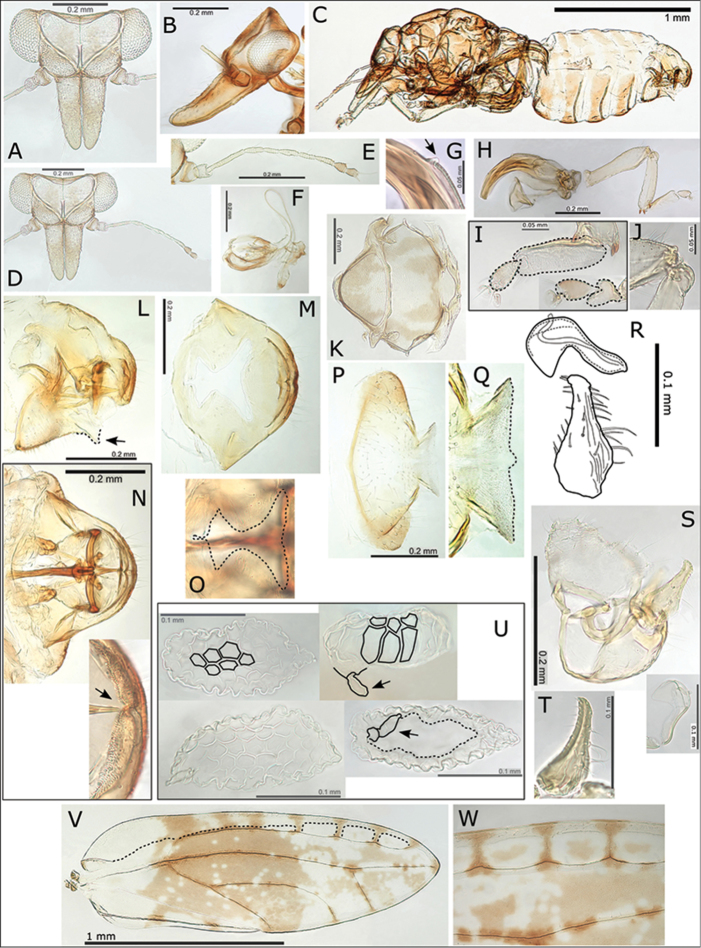
*Swezeyana rubra* sp. n. **A** head **B** head (lateral view) **C** female **D** head and antenna **E** antenna **F** proboscis **G** reduced meracanthus (indicated) **H** hind leg **I** metatarsi (outlined), inset comparative size of mesotarsi (outlined) **J** base of hind tibia **K** dorsum of thorax **L** female terminalia (subgenital beak indicated, apex outlined) **M** female proctiger (dorsal view) **N** female terminalia (dorsal view), inset apex of proctiger (ventral view, cleft indicated) **O** detail of anal ring (outlined) **P** female subgenital plate (ventral view) **Q** detail of posterior apex of female subgenital plate with beak and membrane (outlined) **R** aedeagus and paramere **S** male terminalia, inset aedeagus **T** paramere (posterior view) **U** eggs (semi-hexagonal sculpturing, pedicel and unsculptured underside outlined and indicated) **V** fore wing, with interior edge of ventral margin outlined **W** fore wing detail of pigmented cross veins.

**Adult.** General body colour red or red-brown, last 2-3 antennal segments darker brown. Fore wing distinctly patterned with irregular clouds of red pigmentation, mottled red-brown pattern in apical 2/3 of fore wing, with basal portion either clear (males) or slightly mottled (females), males have a darker almost solid red-brown area of pigmentation across middle of wing membrane, wing veins variably brown to speckled brown with darker brown patches indicating position of cross pseudoveins, intersections of veins and wing margin, as well as two brown patches on the dorsal claval wing margin, and on vein R+M+Cu_1_ just basal to vein trifurcation, there are distinctly unpigmented areas surrounding marginal clusters of radular spines (Fig. 1I). Fore wing apex bluntly acute, shape shorter and broader than other *Swezeyana* (WL:WW < 3.10); pseudopterostigma long (Fig. 8V), 5-7 cross pseudoveins in cell r_1_ (Fig. 8W); surface spinules densely distributed in all cells, but cell c+sc often partly to almost entirely composed of a thickened ventral wing margin (C+Sc); medium long setae on ventral margin and medium short to short setae on veins and dorsal margin. Antennae short (ratio AL:HW 0.93–1.03) (Fig. 8C–D); genal processes relatively short (GP < 0.35 mm, ratio HW:GP > 1.65) and not or slightly upturned at apices (Fig. 8A–C); medium short to short setae on vertex and thorax. Meracanthus small (Fig. 8G), genual spine reduced or absent (Fig. 8J). Male terminalia (Fig. 8R–T): paramere short (ratio PL:HW < 0.25), tapering to slightly anteriorly projecting apex with two short stout setae; distal aedeagus segment short relative to paramere (ratio PL:AEL < 1.10), apex developed into a large rounded hook with acute apex. Female terminalia (Fig. 8L–Q): proctiger dorsal surface strongly convex apically, apex broad, blunt, bearing medial cleft and fringed with stout setae, anal ring medium long (ratio FP:RL 1.71–2.19), with reduced head compartment at proximal end, distal portion of ring margin smooth; subgenital plate slightly convex with little or no medial cleft but with beak and membrane extended (Fig. 8P–Q).

**Egg.** Pale with loosely structured hexagonal sculpturing dorsally (Fig. 8U).

**Immature.** Unknown.

###### Host plant.

*Planchonella sandwicensis*.

###### Distribution.

Hawaii. Only known from PuuWaaWaa area.

###### Etymology.

Named for the generally red body colouration (adjective in the nominative singular).

###### Comments.

This species was collected from the same individual host tree as *S. hawaiiensis* (see comments for *S. hawaiiensis*).

###### Material examined.

Holotype male (slide mounted), PuuWaaWaa, Hawaii, USA, N19.784, W-155.833, 820m, ex *Planchonella sandwicensis*, 29 July 2002, “440B-02” D. Percy leg. (BMNH). Paratypes (slide mounted) 2f, as for holotype (BMNH). Other material: 2m 8f, PuuWaaWaa Forest Reserve, Hawaii, USA, 2600 ft, ex *Planchonella sandwicensis*, 23 February 2011, “JG5B” J. Giffin leg. (BMNH).

###### Gene sequences.

MH001523 (COI) MH001529 (cytB) (JG5B); MG988834 (COI) MG989156 (cytB) (440B-02).

#### Species group: *reticulata*

##### Swezeyana reticulata

Taxon classificationAnimaliaHemipteraTriozidae

Caldwell, 1940

Figures 1G , 9

Swezeyana reticulata Caldwell, 1940: 390.

###### Description.

**Figure 9.**
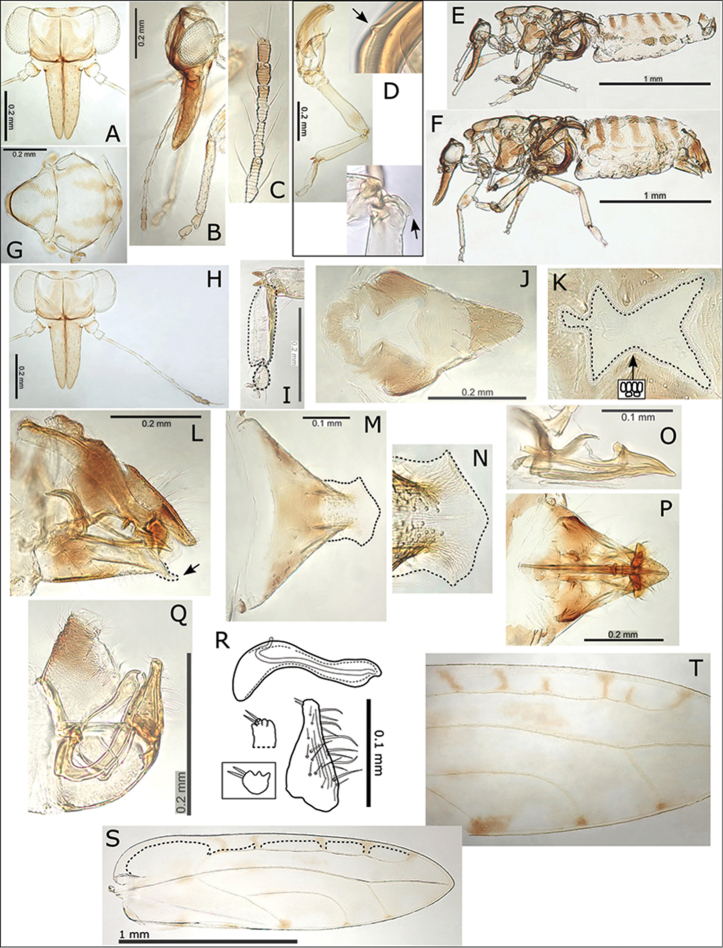
*Swezeyana reticulata*. **A** head **B** head and antenna (lateral view) **C** detail of antenna **D** hind leg, inset highly reduced meracanthus (indicated) and base of hind tibia with genual spine (indicated) **E** male **F** female **G** dorsum of thorax **H** head and antenna **I** metatarsi (outlined) **J** female proctiger (dorsal view) **K** detail of anal ring (outlined), illustrating circumanal ring pores **L** female terminalia (subgenital beak indicated, apex outlined) **M** female subgenital plate (ventral view, apex outlined) **N** detail of posterior apex of female subgenital plate with beak and membrane (outlined) **O** ovipositor **P** female terminalia (dorsal view), lateral valves extending beyond proctiger **Q** male terminalia **R** aedeagus and paramere, with details of paramere apex (above interior view, below dorsal view) **S** fore wing, with interior edge of ventral margin outlined **T** fore wing detail of pigmented cross veins.

**Adult.** General body colour yellow-brown to darker brown, last 2-3 antennal segments darker brown. Fore wing patterned with irregular clouds of brown pigmentation (although less distinctly than in *S. rubra* and *S. tentaculata*), darker brown patches indicate position of cross pseudoveins, intersections of veins and wing margin, as well as 1-2 brown patches on the dorsal claval wing margin, and a more or less distinct patch on vein R+M+Cu_1_ just basal to vein trifurcation, unpigmented areas surround the marginal clusters of radular spines (Fig. 1G). Fore wing apex acute to bluntly acute; pseudopterostigma short (Fig. 9S), 3-4 cross pseudoveins in cell r_1_ (Fig. 9T); surface spinules densely distributed in all cells; medium long setae on ventral margin and short setae on veins and dorsal margin. Antennae medium short (ratio AL:HW 1.25–1.40) (Fig. 9B, I); genal processes medium long (GP < 0.40 mm, ratio HW:GP < 1.65) and slightly upturned at apices (Fig. 9A–B, E–F); medium short to short setae on vertex and thorax. Meracanthus small, genual spine developed (Fig. 9D). Male terminalia (Fig. 9Q–R): paramere short (ratio PL:HW < 0.25), tapering to apex with two short stout setae; distal aedeagus segment long relative to paramere (ratio PL:AEL < 0.80), apex developed into a large rounded hook with bluntly acute apex. Female terminalia (Fig. 9J–P): proctiger dorsal surface more or less straight, tapering to bluntly acute apex without medial cleft, anal ring relatively short (ratio FP:RL 2.17–2.45), with well-developed head compartment at proximal end, distal portion of ring margin slightly convoluted; subgenital plate more or less straight ventrally with little or no medial cleft and with beak and membrane slightly extended (Fig. 9L–N).

**Egg.** Pale with shallow hexagonal indentations dorsally.

**Immature.** Colour: Mottled, cream and red-brown. Structure: 5^th^ instar with circumanal ring wide, and more or less straight, with a single row of uninterrupted elongate cells (Fig. 13G). Chaetotaxy: 5^th^ instar with marginal, narrow, blunt sectasetae, and sub-marginal longer acute, simple setae on enlarged, ridged annuli or pediments (Fig. 13A–C); dorsal surface rugose, with small acute, simple setae on bulbous, ridged pediments, and with distinct arrangement of 28 protruding tubercles (13 on head and thorax, 15 on abdomen, of these 10 are medial, and 18 are lateral or sub-marginal), tubercles bearing scattered small, blunt, simple setae (Fig. 13D–F). 1^st^ instar with marginal narrow, blunt sectasetae (Fig. 12A); 2^nd^ instar with marginal narrow, blunt sectasetae, sub-marginal longer acute simple setae on enlarged and ridged annuli or pediments, and 7 sub-marginal tubercles, with 3 on thorax bearing 1-2 acute simple setae, and a marginal sub-apical pair of long simple setae on abdomen (Fig. 12B–D); 3^rd^ – 4^th^ instar chaetotaxy and tubercle arrangement similar to 5^th^ instar (Fig. 12E–F). In 4^th^ and particularly 5^th^ instar, 4 of the dorsal tubercles are more darkly pigmented (these are the distal medial tubercles on thorax and abdomen, and the proximal lateral tubercles on abdomen; Fig. 13E–F).

**Immature measurements (mm) and ratios**: 5^th^ instar (n = 4): BL 1.67–1.71; BW 1.06–1.15; WPL 0.85–0.88; CPL 0.73–0.79; CPW 0.62–0.97; RW 0.17–0.18; HW 0.52–0.57; AL 0.19–0.20; BL:BW 1.49–1.57; HW:AL 2.60–2.96; CPW:RW 3.70–5.77.

###### Host plant.

*Planchonella sandwicensis*.

###### Distribution.

Kauai (possibly also on Maui, see comments). Appears to be the most common of the three *Swezeyana* species found on Kauai.

###### Comments.

Tuthill (1966) notes the co-occurrence of two species on Maui, and refers to these as *S. elongagena* and *S. reticulata*; but *S. reticulata* is here considered endemic to Kauai and the observed species on Maui may be an as yet undescribed species. The immatures as noted by Tuthill have “a remarkable armament of large proturberances [sic] on the dorsal surface” and therefore fit within the *reticulata* group.

###### Material examined.

Holotype female (BPBM, not located). Other material: 1f, Nualolo Trail, Kokee State Park, Kauai, USA, on *Polyscias waimeae*, 25 May 2002, “370-02” D. Percy leg. (BMNH). 10m 4f 7i, Kokee State Park, Kauai, USA, N22.1444, W-159.6477, ex *Planchonella sandwicensis*, 29 October 2005, “Hi01-05” D. Percy leg. (BMNH). 5m 1f, Kokee State Park, Kauai, USA, N22.1503, W-159.6453, ex *Planchonella sandwicensis*, 29 October 2005, “Hi02-05” D. Percy leg. (BMNH). 12m 7f, Kokee State Park, Kauai, USA, N22.1309, W-159.6388, ex *Planchonella sandwicensis*, 30 October 2005, “Hi05-05” D. Percy leg. (BMNH). 1m 10f, Kokee State Park, Kauai, USA, N22.0948, W-159.6953, ex *Planchonella sandwicensis*, 30 October 2005, “Hi11-05” D. Percy leg. (BMNH).

###### Gene sequences.

MG988833 (COI) MG989154 (cytB) (Hi01-05); MH001524 (COI) MH001530 (cytB) (Hi05-05 adult); MH001525 (COI) MH001531 (cytB) (Hi05-05 immature); MH001526 (COI) MH001532 (cytB) (Hi11-05).

##### Swezeyana tentaculata sp. n.

Taxon classificationAnimaliaHemipteraTriozidae

http://zoobank.org/6605B22E-FEFD-4EA5-A501-5E2D094A877A

Figures 1H , 10

###### Diagnosis.

Medium sized, red-brown species, with fore wing membrane patterned, antennae medium long, genal processes long, paramere short, and female proctiger more or less straight dorsally.

###### Description.

**Figure 10.**
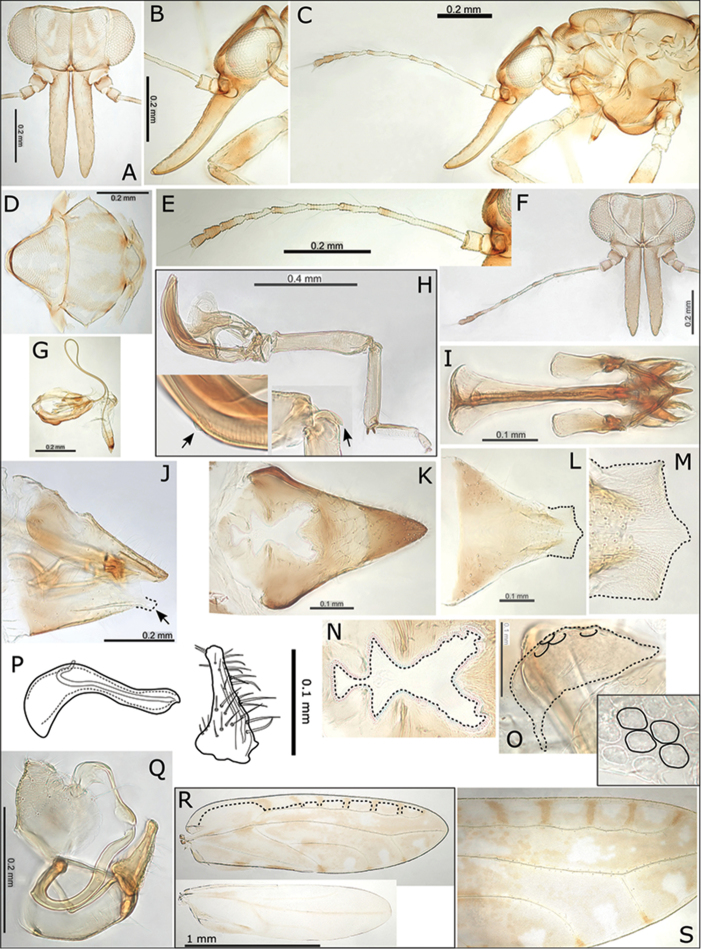
*Swezeyana tentaculata* sp. n. **A** head **B** head (lateral view) **C** head and antenna (lateral view) **D** dorsum of thorax **E** antenna **F** head and antenna **G** proboscis **H** hind leg, inset highly reduced meracanthus (indicated) and base of hind tibia with genual spine (indicated) **I** female terminalia endoskeleton (dorsal view) **J** female terminalia (subgenital beak indicated, apex outlined) **K** female proctiger (dorsal view) **L** female subgenital plate (ventral view, apex outlined) **M** detail of posterior apex of female subgenital plate with beak and membrane (outlined) **N** detail of anal ring (dorsal view, outlined) **O** egg (outlined), inset detail of sculpturing on dorsal service **P** aedeagus and paramere **Q** male terminalia **R** fore wing (above), with interior edge of ventral margin outlined, hind wing (below) **S** fore wing detail of pigmented cross veins and unpigmented membrane surrounding marginal radular spine clusters.

**Adult.** General body colour red to red-brown, last 2-3 antennal segments darker brown. Fore wing distinctly patterned with irregular clouds of red-brown pigmentation, darker brown patches indicate position of cross pseudoveins, intersections of veins and wing margin, as well as two brown patches on the dorsal claval wing margin, and on vein R+M+Cu_1_ just basal to vein trifurcation, there are distinctly unpigmented areas surrounding marginal clusters of radular spines (Figs 1H, 10S). Fore wing apex bluntly acute; pseudopterostigma short (Fig. 10R), 4-6 cross pseudoveins in cell r_1_ (Fig. 10R–S); surface spinules densely distributed in all cells; long setae on ventral margin and medium short to short setae on veins and dorsal margin. Antennae medium long (ratio AL:HW 1.40–1.61) (Fig. 10C, E–F); genal processes long (GP ≥ 0.35 mm, ratio HW:GP ≤ 1.35) and upturned at apices (Figs 10A–C); medium short to short setae on vertex and thorax. Meracanthus reduced, almost absent, genual spine developed (Fig. 10H). Male terminalia (Fig. 10P–Q): paramere short (ratio PL:HW < 0.30), tapering to apex with two short stout setae; distal aedeagus segment long relative to paramere (ratio PL:AEL < 0.90), apex developed into a large rounded hook with acute apex. Female terminalia (Fig. 10I–N): proctiger dorsal surface more or less straight, tapering to bluntly acute apex without medial cleft, anal ring relatively short (ratio FP:RL 2.24–2.59), with well-developed head compartment at proximal end, distal portion of ring margin convoluted; subgenital plate more or less straight ventrally with little or no medial cleft and with beak and membrane slightly extended (Fig. 9L–N).

**Egg.** Pale, sculpturing consisting of rounded indentations dorsally (Fig. 10O).

**Immature.** Colour: Mostly red-brown, some cream mottling. Structure: 5^th^ instar with circumanal ring wide, slightly constricted medially and lateral apices upturned, with a single row of uninterrupted elongate cells (Fig. 13I). Chaetotaxy: 5^th^ instar with marginal, pointed sectasetae, and sub-marginal longer acute, simple setae on enlarged, ridged annuli or pediments (Fig. 13H, J–K); dorsal surface rugose, with small club setae, and with distinct arrangement of 28 protruding tubercles and tentacles (13 on head and thorax, 15 on abdomen, of these 10 are medial, and 18 are lateral or sub-marginal), all but 4 of the dorso-medial protuberances (on abdomen) are developed into elongate tentacles bearing scattered small, blunt, simple setae on the apical portion, and small club setae on the basal portion, tentacle apices bear 1-2 acute simple setae (Fig. 13L). 3^rd^ – 4^th^ instar chaetotaxy and tubercle/tentacle arrangement similar to 5^th^ instar (Fig. 12G–J); in 3^rd^ instars the lateral and submarginal tubercles bear 2 club setae at the apices (Fig. 12I), and the dorso-medial tubercles destined to become tentacles are larger and bear several club setae (Fig. 12H), by 4^th^ instar elongation into tentacles is already evident. Somewhat more darkly pigmented tentacles are found in the same position as described for *S. reticulata* (the distal medial tentacles on thorax and abdomen, and the proximal lateral tentacles on abdomen; Fig. 13H, K). The tentacles are also evident in photographs of immatures on the leaf surface (Fig. N).

**Immature measurements (mm) and ratios**: 5^th^ instar (n = 3): BL 1.48–1.52; BW 0.91–0.97; WPL 0.73–0.76; CPL 0.64; CPW 0.76; RW 0.16–0.17; HW 0.48–0.52; AL 0.18; BL:BW 1.56–1.63; HW:AL 2.61–2.95; CPW:RW 4.51–4.92.

###### Host plant.

*Planchonella sandwicensis*.

###### Distribution.

Kauai. Only known from Kokee State Park.

###### Etymology.

Named for the distinctly long tentacles on the dorsum of immatures (adjective in the nominative singular).

###### Comments.

Found sympatrically with *S. reticulata* and *S. elongagena* on the same individual plants. Immatures were observed among the ferugineous trichomes on the undersides of leaves, often along the leaf mid-rib (Fig. 13N).

###### Material examined.

Holotype male (slide mounted), Kokee State Park, Kauai, USA, N22.1444, W-159.6477, ex *Planchonella sandwicensis*, 29 October 2005, “Hi01-05” D. Percy leg. (BMNH). Paratypes (slide mounted) 3f 7i, as for holotype (BMNH). Other material: 2m 2f, Kokee State Park, Kauai, USA, N22.1309, W-159.6388, ex *Planchonella sandwicensis*, 30 October 2005, “Hi05-05” D. Percy leg. (BMNH).

###### Gene sequences.

MG989157 (cytB) (Hi01-05).

#### Species group: unplaced

##### Swezeyana magnaccai sp. n.

Taxon classificationAnimaliaHemipteraTriozidae

http://zoobank.org/5DA970DA-2E09-495A-9EF0-9654946E5348

Figures 1F , 11

###### Diagnosis.

Small, red- to yellow-brown species, with fore wing membrane patterned, antennae and genal processes medium long, and paramere short.

###### Description.

**Figure 11.**
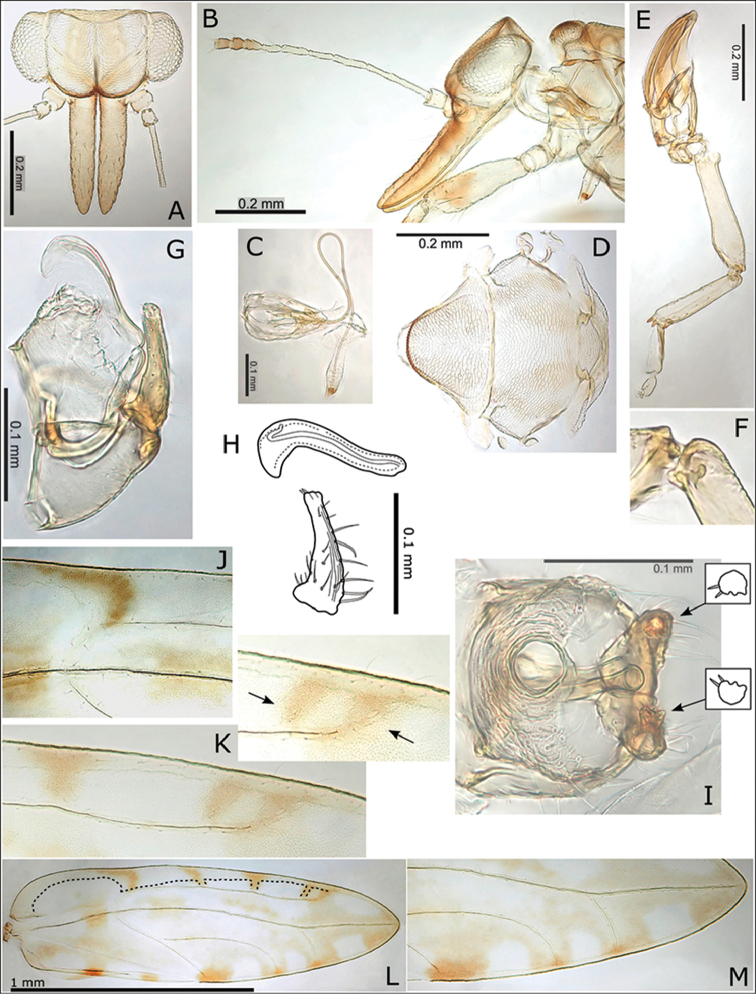
*Swezeyana magnaccai* sp. n. **A** head **B** head and antenna (lateral view) **C** proboscis **D** dorsum of thorax **E** hind leg **F** base of hind tibia **G** male terminalia **H** aedeagus and paramere **I** male terminalia (dorsal view), inset details of paramere apices **J** fore wing detail of termination of vein R at base of pseudopterostigma **K** fore wing detail of incomplete termination of vein Rs at wing margin (inset incomplete veins indicated) **L** fore wing, with interior edge of ventral margin outlined **M** fore wing detail of unpigmented membrane surrounding marginal radular spine clusters.

**Adult.** General body colour orange-red to yellow-brown, last 2-3 antennal segments darker brown. Fore wing patterned with irregular clouds of orange-brown pigmentation, darker patches indicate position of cross pseudoveins, intersections of veins and wing margin, as well as 1-2 darker patches on the dorsal claval wing margin, and a more or less distinct patch on vein R+M+Cu_1_ just basal to vein trifurcation, unpigmented areas surround the marginal clusters of radular spines (Figs 1F, 11M). Fore wing apex bluntly acute; pseudopterostigma relatively long (Fig. 11J, L), 2-3 cross pseudoveins in cell r_1_ (Fig. 11L); surface spinules densely distributed in all cells; medium long setae on ventral margin and medium short to short setae on veins and dorsal margin. Antennae medium long (ratio AL:HW 1.41) (Fig. 11B); genal processes medium long (GP < 0.35 mm, ratio HW:GP < 1.65) and not or only slightly upturned at apices (Fig. 11A–B); medium short to short setae on vertex and thorax. Meracanthus extremely small to almost absent, genual spine reduced or absent (Fig. 11E–F). Male terminalia (Fig. 11H–I): paramere short (ratio PL:HW < 0.30), tapering to apex with two short stout setae; distal aedeagus segment long relative to paramere (ratio PL:AEL < 1.10), apex developed into a large rounded hook with acute apex.

**Figure 12.**
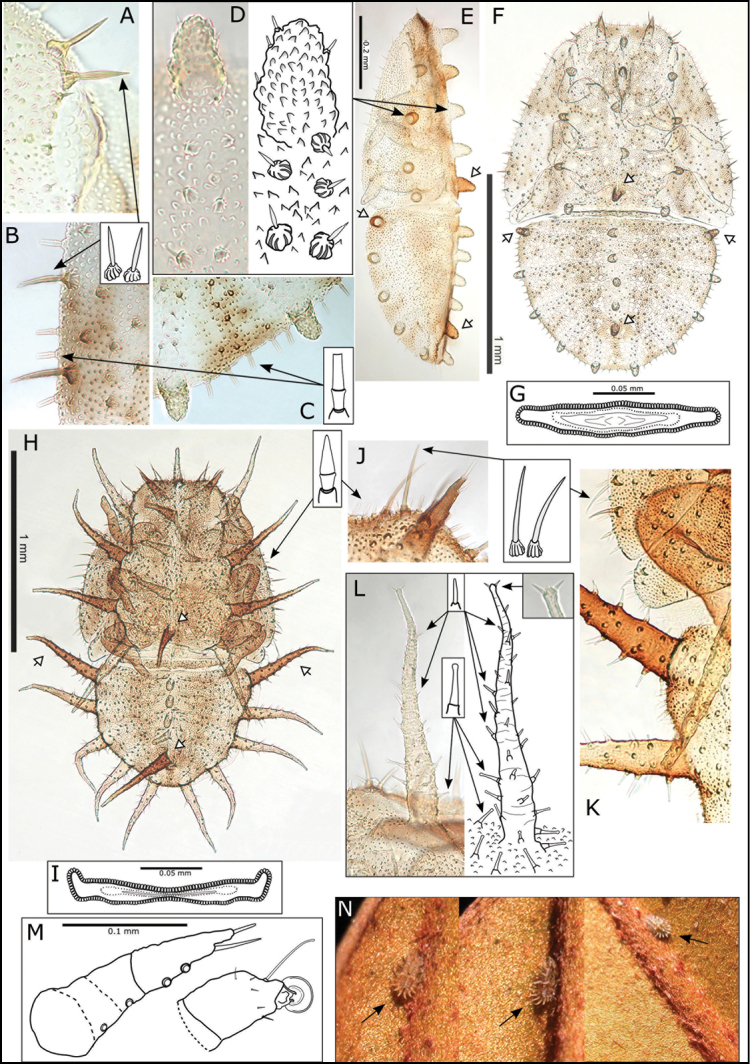
*Swezeyana reticulata* and *Swezeyana tentaculata* 5^th^ instar immatures. **A–G, M** *S. reticulata*: **A** detail of dorsal lanceolate setae with inflated and ridged bases anterior of eye **B** detail of dorsal sub-marginal lanceolate setae with inflated and ridged bases on margin of wing pads **C** detail of tubercles on margin of abdomen, and marginal narrow, blunt sectasetae **D** detail of dorsal tubercle bearing small simple setae, and small lanceolate setae with greatly inflated and ridged bases on surrounding surface **E** lateral view showing arrangement of dorsal tubercles, open arrows indicate position of thoracic and abdominal tubercles typically more darkly pigmented **F** dorsal view, open arrows indicate position of thoracic and abdominal tubercles typically more darkly pigmented **G** anal ring **H–L, N** *S. tentaculata*: **H** dorsal view, open arrows indicate position of thoracic and abdominal tentacles typically more darkly pigmented, inset detail of marginal pointed sectasetae **I** anal ring **J, K** detail of dorsal sub-marginal long, simple setae with narrowly inflated and ridged bases, **K** also shows different pigmentation for 1^st^ and 2^nd^ tentacle on abdomen margin **L** detail of long tentacle with simple setae towards the apex and a pair of small simple setae apically, and longer slightly capitate rod setae towards the base and on surrounding surface **M** tarsus and antenna (similar for both species) **N** red-brown *S. tentaculata* immatures found along the midribs on undersides of leaves among the red-brown leaf trichomes.

**Figure 13.**
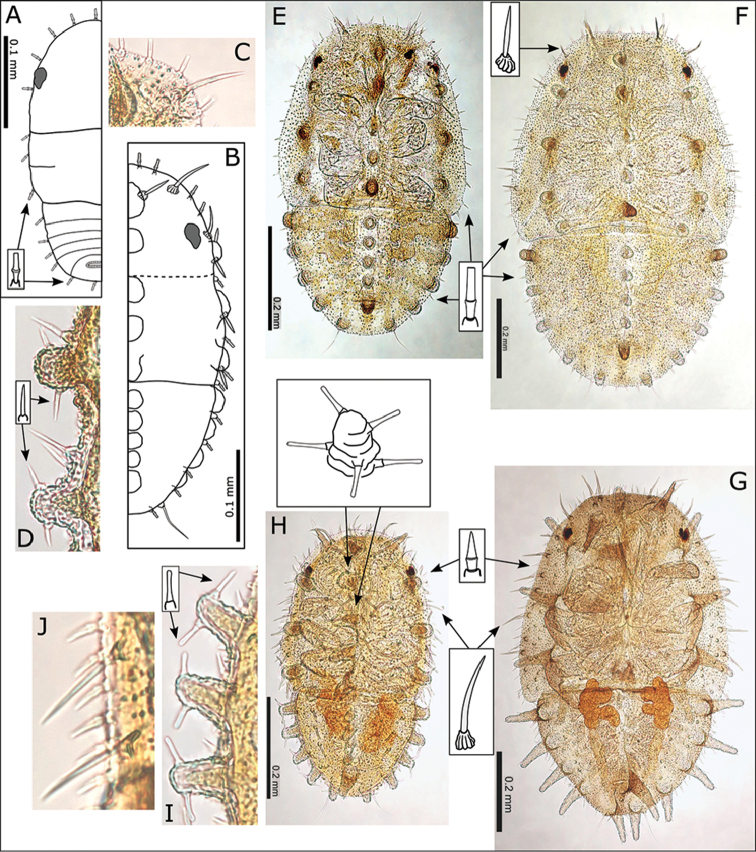
*Swezeyana reticulata* and *Swezeyana tentaculata* 1^st^-4^th^ instar immatures. **A–F** *S. reticulata*: **A** 1^st^ instar, inset detail of marginal narrow, blunt sectasetae **B** 2^nd^ instar with appearance of tubercles **C** detail of 2^nd^ instar anterior marginal and sub-marginal head setae **D** detail of 2^nd^ instar marginal tubercles on the thorax bearing simple setae towards apices **E** 3^rd^ instar, inset detail of marginal narrow, blunt sectasetae **F** 4^th^ instar, inset detail of dorsal and sub-marginal lanceolate setae with inflated and ridged bases **G–J** *S. tentaculata*: **G** 4^th^ instar, with details of marginal pointed sectasetae, and dorsal and sub-marginal simple setae with narrowly inflated and ridged bases **H** 3^rd^ instar, with detail of larger dorso-medial tubercles bearing spiral of slightly capitate rod setae; marginal pointed sectasetae, and dorsal and sub-marginal simple setae with narrowly inflated and ridged bases as for 4^th^ instar **I** detail of 3^rd^ instar pairs of slightly capitate rod setae near the apices of marginal tubercles on the abdomen **J** detail of 3^rd^ instar marginal pointed sectasetae, and dorsal sub-marginal simple setae.

**Egg.** Unknown.

**Immature.** Unknown.

###### Host plant.

*Planchonella sandwicensis*.

###### Distribution.

Oahu. Only known from the Waianae Mountains.

###### Etymology.

Named for Karl Magnacca, a talented biologist who contributed several specimens for this study (noun in the genitive case).

###### Comments.

Currently only known from males, it may belong in the *reticulata* species group based on fore wing characters and paramere shape, but is currently unplaced.

###### Material examined.

Holotype male (slide mounted), Mokuleia Forest Reserve, Pahole, N Waianae Mnts, Oahu, USA, N21.53208, W-158.1786, ex *Planchonella sandwicensis*, 6 July 2014, “Hi74-14” D. Percy leg. (BMNH). Paratypes (slide mounted) 2m, as for holotype (BMNH). Other material: 1m, Puu Hapapa, Central Waianae Mnts, Oahu, USA, N21.4666, W-158.1029, ex *Planchonella sandwicensis*, 17 May 2014, “KM14-14” K. Magnacca leg. (BMNH).

###### Gene sequences.

KY294144 (COI) KY294628 (cytB) (Hi74-14) [previously submitted to GenBank as *Swezeyana reticulata* Caldwell, 1940 (in Percy 2017a)]; KY294145 (COI) KY294629 (cytB) (KM15-14) [previously submitted to GenBank as *Swezeyana reticulata* Caldwell, 1940 (in Percy 2017a)].

## Discussion

All *Swezeyana* species are hosted by a single, endemic Hawaiian host plant, *Planchonella sandwicensis* (Sapotaceae); and this long lived woody plant is distributed across all major islands in the archipelago. Co-occurrence of two or more *Swezeyana* species collected from the same tree are known for at least four islands, Kauai, Oahu, Maui, and Hawaii (Caldwell 1940, Tuthill 1966, and this study). In reference to two species found on Maui, Tuthill (1966) remarked that “the occurrence of the two distinct species in the same ecological niche is unusual”, and this is a striking pattern in the genus. Expectations are that, in close sympatry, specialization for different niches on the host plant will, at some scale, contribute to driving diversification, particularly if the species represent sympatric speciation (Matsubayashi et al. 2010). The morphological, ecological and molecular evidence for sympatric speciation in *Swezeyana* provides some interpretive challenges.

Supporting the possibility of sympatric speciation is the single island endemism of most *Swezeyana* species that are known to co-occur, as well as some support from the molecular data. Due to a lack of backbone resolution in the phylogeny based on the two region DNA barcode data, short branches and variably supported nodes for the three putative same island species pairs, indicate these two gene regions are most effective for confirmation of species assignment, but less effective for resolving relationships between species. Nevertheless, of the three co-occurring species pairs, those on Oahu and Hawaii (*S. atra* and *S. oahuensis* on Oahu; and *S. hawaiiensis* and *S. rubra* on Hawaii) represent putative sister pairs, while a species pair on Kauai (*S. reticulata* and *S. tentaculata*) provides an unequivocal example (with stronger phylogenetic support likely due to a more recent speciation event) for a sister taxon relationship for these co-occurring species. The Kauai species pair have repeatedly been found co-occurring on the same individual leaves and both species are likely endemic to Kauai. Therefore, in the Kauai example, geography and molecular data provide strong evidence for diversification in sympatry.

The Hawaiian psyllids in the genus *Pariaconus* that feed on *Metrosideros polymorpha* also show similar evidence of divergence in sympatry, but these *Metrosideros*-feeding species often exhibit clear niche partitioning of the host species by different galling and non-galling biologies and/or occupation of different parts of the plant and/or different plant morphotypes (Percy 2017a). In contrast, *Swezeyana* species, as far as we know, all have free-living immatures found on the leaf surface, and there are no clearly apparent biological or microhabitat shifts coincident with occupying or partitioning the host plant. Most studies that have looked at species pairs in ecologically equivalent systems (e.g., same host plant) have concluded that in the absence of ecologically driven divergence (e.g., host races), allopatry must be important for either incomplete or complete non-ecological speciation (Jordal et al. 2006, Nyman et al. 2010). There is no evidence for past allopatry in co-occurring *Swezeyana*, but within island microgeographic allopatry may or may not have been more prevalent in the past when there was less forest disturbance, and more abundance of *Planchonella* trees.

Other than the sister taxon pair on Kauai, all *Swezeyana* species sampled for the DNA analysis are highly divergent from one another, and the lack of backbone resolution in the genus suggests there may have been a modest but rapid early radiation after colonization of the Hawaiian Islands, with little more recent divergence. Alternatively, more recent divergence events may have been obscured by extinction, or not yet discovered. Molecular divergence provides a comparison with other Hawaiian psyllid lineages, and suggests that *Swezeyana*, is another relatively old endemic genus in the Hawaiian Islands (Percy 2017a,b). Maximum pairwise molecular distances within *Swezeyana*: 19.9%, can be compared with *Hevaheva* Kirkaldy, 1902: 17.5%, *Pariaconus*: 16.9%, *Stevekenia* Percy, 2017: 16.8%, and *Hemischizocranium* Tuthill, 1956: 13.9% (Fig. 2). In addition, it is interesting to compare maximum intraspecific divergence for two widespread non-Hawaiian taxa, *Trioza urticae* (Linné, 1758) (the common nettle psyllid): 2.8%, sampled from across Europe (southern Greece to arctic Norway, ~3500 km) (Wonglersak et al. 2017), and *Mesohomotoma hibisci*: 13.7%, a Pacific-wide species sampled from Singapore, New Caledonia, and French Polynesia (~7000 km and ~4500 km) (Percy 2017a); these two extremely widespread taxa are distributed over similarly large distances, but one is an almost entirely terrestrial landscape, whereas the other is an oceanic island landscape. The data indicates there is surprisingly efficient dispersal over large terrestrial distances (in this case facilitated by a highly abundant host plant, the common nettle, *Urtica dioica*), and in contrast relatively poor dispersal across oceans (despite the host plant, in this instance, *Hibiscus tiliaceus*, being abundant in nearly all Pacific regions). The different challenges to dispersal and gene flow across these landscapes are the most likely cause of the different levels of intraspecific molecular divergences observed, and similar processes (i.e., reduced dispersal and gene flow) probably also contribute to the high degree of single island endemism generally found in the Hawaiian psyllid fauna. Understanding the role of varying genetic landscapes in newly forming species and island speciation has been exemplified in studies of Darwin’s finches in the Galapagos (Petren et al. 2005, Han et al. 2017), but obtaining adequate sampling to test similar hypotheses in psyllids will always be challenging.

Recognition of the two species groups in *Swezeyana* (*elongagena* group and *reticulata* group) is based primarily on the strikingly different structures of the female terminalia. This morphology is characterized by different development of the apodemes giving rise to distinct endoskeletons in the two species groups, and these different female terminalia structures imply traits related to oviposition that could be important in diversification, although the ovipositor itself is more or less invariant. However, because taxa in different species group, and taxa in the same species group, co-occur on the same individual plants, seemingly without any observable niche partitioning, it is particularly difficult to understand what may have initially driven this striking differentiation in female terminalia structure. Although the two species groups are recovered in the molecular topology, there is little to no bootstrap support for the *elongagena* group, emphasizing again the effectiveness of these barcode regions for identifying individuals to species but not for providing resolution at deeper nodes for higher classification. Nevertheless, the molecular data does serve to emphasise that the presence and number of cross veins and wing patterning varies within species groups. For instance, the two species with the greatest degree of wing patterning, as well as highest number of cross veins between Rs and the wing margin, *S. rubra* and *S. tentaculata*, are in different species groups, *elongagena* group and *reticulata* group respectively, and the more patterned wings with a greater number of cross veins appear to have resulted from convergence in these characteristics. A wing type with numerous cross veins may be the ancestral state, but at present more sampling and analysis are required to test this hypothesis. Some of the fore wing and other unusual morphological traits in the genus may point to different dimensions of niche specialization among species. For instance, the often striking reddish to pink colouration/highlights found in many *Swezeyana* may provide camouflage against the rusty coloured trichomes on the host plant leaves, and variation in body and wing colouration may be involved in different strategies for predator avoidance or mate selection; the notably thickened anterior fore wing margin is another unusual characteristic of the genus that is as yet unexplained, but may play a role in acoustic communication (Percy et al. 2006). Other unusual characters such as the extremely long genal processes and long basal metatarsi are likewise unexplained. Variation in both colour and communication traits may represent additional niche dimensions (Jiggins 2008, Nosil and Sandoval 2008) in which selection and adaptation could have driven divergence in these species. The strikingly different immature morphology among co-occurring species also suggests that, despite no observable niche partitioning, immature morphologies may have arisen in response to different selection pressures, and therefore immatures may indeed be utilizing the host environment differently.

*Swezeyana* species are not generally abundant, for instance in comparison to some of the *Metrosideros*-feeding *Pariaconus* (Percy 2017a). During this study, typically not more than a handful of *Swezeyana* individuals were captured from one location/tree, although Tuthill (1966) recorded the two Maui species as locally abundant, as did Swezey (1954) for *S. elongagena* on Maui. Generally low abundance may partly be due to the scattered distribution of *Planchonella* trees, which are less common than *Metrosideros*. In addition, there is an increasing lack of new regeneration of *Planchonella*, which may preclude the establishment and growth of larger psyllid populations. However, it is possible sampling bias plays a role because large *Planchonella* trees are not easily accessible and the new leaf growth is mostly in the canopy. In addition, *Planchonella sandwicensis*, as with much of the native Hawaiian flora, was likely much more abundant, but habitat disturbance and clearing have impacted both plant and host specific taxa such as psyllids (Percy 2017b).

The taxonomic affiliations of *Swezeyana* to other genera within the family Triozidae is uncertain due to the lack of a robust phylogenetic framework for the family, but it is worth noting that the immatures of *Hemischizocranium*, which are also free-living on the leaf surface, have a distinct medial (anterior-posterior) linear row of small dorsal tubercles (Tuthill 1956), similar in size to those illustrated here for 3^rd^ instars of *S. reticulata*. Furthermore, in a recent phylogenomic analysis, using the most comprehensive sampling to date of the Psylloidea (Percy *et al*. 2018), a major clade of predominantly non-galling triozid species includes three Hawaiian endemic genera *Swezeyana, Hemischizocranium*, and *Stevekenia* Percy, 2017. Additional sampling within this clade would be useful in determining the relationships of these genera and their source origins outside the Hawaiian Islands.

## Supplementary Material

XML Treatment for Swezeyana

XML Treatment for Swezeyana elongagena

XML Treatment for Swezeyana atra

XML Treatment for Swezeyana hawaiiensis

XML Treatment for Swezeyana magna

XML Treatment for Swezeyana oahuensis

XML Treatment for Swezeyana rubra

XML Treatment for Swezeyana reticulata

XML Treatment for Swezeyana tentaculata

XML Treatment for Swezeyana magnaccai
